# O Complexo Quebra-Cabeça do Fenótipo Hipertrófico: Uma Abordagem Prática para o Clínico

**DOI:** 10.36660/abc.20240529

**Published:** 2025-03-18

**Authors:** Alex dos Santos Felix, Silvio Henrique Barberato, Marcelo Dantas Tavares de Melo, Sílvia Aguiar Rosa, Nuno Cardim

**Affiliations:** 1 Instituto Nacional de Cardiologia Rio de Janeiro RJ Brasil Instituto Nacional de Cardiologia, Rio de Janeiro, RJ – Brasil; 2 DASA - Diagnósticos da América SA Rio de Janeiro RJ Brasil DASA - Diagnósticos da América SA, Rio de Janeiro, RJ – Brasil; 3 Universidade do Estado do Rio de Janeiro Rio de Janeiro RJ Brasil Universidade do Estado do Rio de Janeiro, Rio de Janeiro, RJ – Brasil; 4 Complexo Hospitalar Américas - Vitória Samaritano Barra Rio de Janeiro RJ Brasil Complexo Hospitalar Américas - Vitória / Samaritano Barra, Rio de Janeiro, RJ – Brasil; 5 CardioEco Centro de Diagnóstico Cardiovascular Curitiba PR Brasil CardioEco Centro de Diagnóstico Cardiovascular, Curitiba, PR – Brasil; 6 Quanta Diagnóstico - Ecocardiografia Curitiba PR Brasil Quanta Diagnóstico - Ecocardiografia, Curitiba, PR – Brasil; 7 Universidade Federal da Paraíba João Pessoa PB Brasil Universidade Federal da Paraíba, João Pessoa, PB – Brasil; 8 Hospital of Santa Marta Lisboa Portugal Hospital of Santa Marta, Lisboa – Portugal; 9 NOVA Medical School Lisboa Portugal NOVA Medical School, Lisboa – Portugal; 10 Hospital CUF Descobertas Lisboa Portugal Hospital CUF Descobertas, Lisboa – Portugal

**Keywords:** Hipertrofia Ventricular Esquerda, Diagnóstico, Diagnóstico Diferencial, Técnicas de Imagem Cardíaca, Testes de Função Cardíaca

## Abstract

A hipertrofia ventricular esquerda (HVE) é frequentemente observada na prática clínica. No entanto, o fenótipo hipertrófico é uma manifestação comum de diversas condições, representando, portanto, um enigma para os médicos em termos de diagnóstico. Diferenciar as etiologias da HVE é fundamental para a tomada de decisão terapêutica, pois diferentes abordagens devem ser implementadas para condições distintas, como HVE secundária a alterações de carga, cardiomiopatia hipertrófica (CMH) ou mimetizadores de CMH.

Em alguns casos, um diagnóstico incorreto ou tardio pode levar à progressão da doença de base com perda adicional da capacidade funcional, alta morbidade e mortalidade.

O uso racional da multimodalidade de imagem cardiovascular é de extrema importância quando são realizados em conjunto com uma avaliação clínica completa e correlacionados com os achados eletrocardiográficos, fornecendo pistas para preencher lacunas. Na maioria das vezes, os exames de imagem são a peça que falta para resolver esse quebra-cabeça desafiador.

Uma abordagem integral é de suma importância na avaliação desses pacientes, pois, muitas vezes, são acompanhados por diversas especialidades, com manifestações sistêmicas variadas. Embora seja necessária uma equipe multidisciplinar para um acompanhamento otimizado desses pacientes, o agente mais importante nessa jornada é o clínico, cuja missão é reunir todos os sinais de alerta e coordenar todos os dados para um diagnóstico assertivo.

O objetivo desta revisão é fornecer uma metodologia pragmática, destacando pistas importantes para discriminar as diversas condições que resultam em HVE.

## Graphical abstract

**Figura Central:**
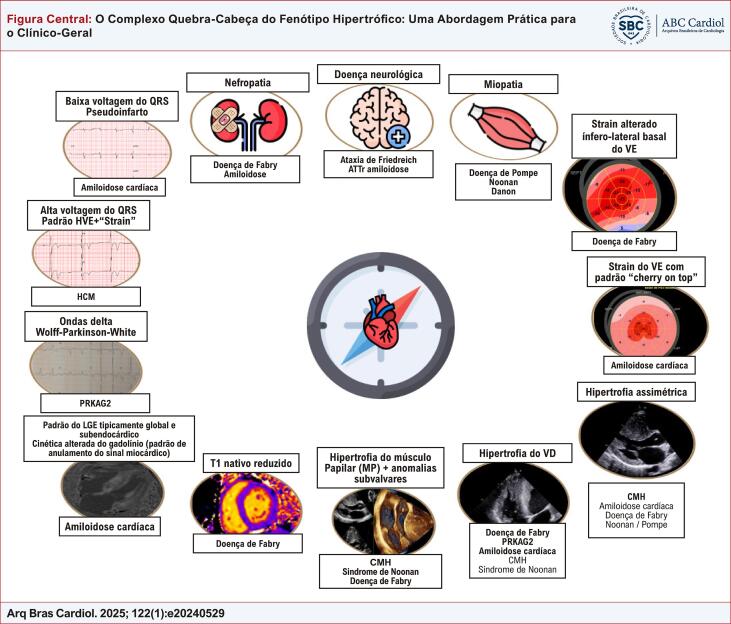
O Complexo Quebra-Cabeça do Fenótipo Hipertrófico: Uma Abordagem Prática para o Clínico-Geral O diagnóstico correto de um paciente com fenótipo hipertrófico do ventrículo esquerdo depende de uma abordagem integrativa, usando pistas da história clínica, exame físico e uso de imagens multimodais. VE: ventrículo esquerdo; HVE: hipertrofia ventricular esquerda; CMH: cardiomiopatia hipertrófica; LGE: realce tardio com gadolínio; VD: ventrículo direito.

## Introdução

**Figura 1:**
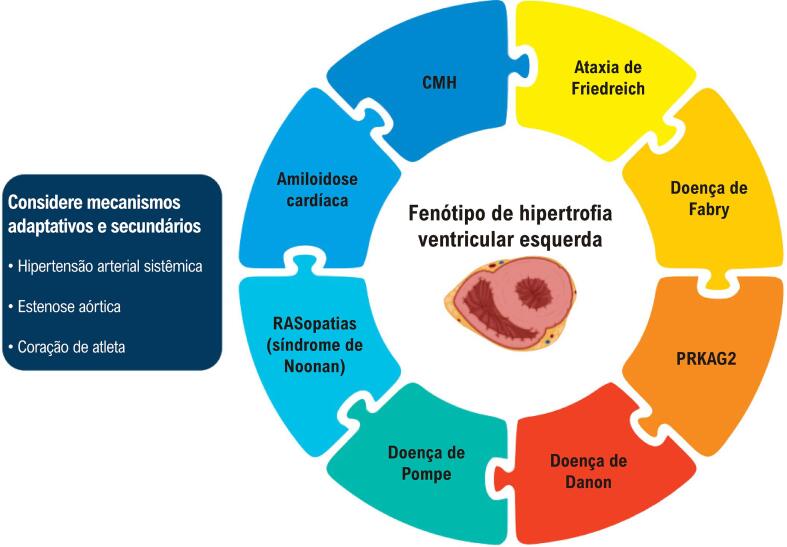
– Quebra-cabeça desafiador do diagnóstico diferencial em pacientes com fenótipo hipertrófico do ventrículo esquerdo.

A hipertrofia ventricular esquerda (HVE) consiste no aumento da espessura da parede do ventrículo esquerdo (VE), sendo frequentemente observada na prática clínica. No entanto, o fenótipo hipertrófico é uma manifestação comum de diversas condições, representando, portanto, um enigma para os médicos em termos de diagnóstico.^1^ A diferenciação entre as etiologias da HVE (Figura 1) é fundamental para a elaboração de abordagens precisas de tratamento. A HVE frequentemente se origina de mecanismos adaptativos secundários, como hipertensão arterial (HA), estenose aórtica (EA) e coração de atleta, ou de diversos outros estados patológicos, abrangendo doenças genéticas e adquiridas, que podem existir simultaneamente. A cardiomiopatia hipertrófica (CMH) é caracterizada pelo espessamento da parede do VE (≥ 15 mm em qualquer parte do VE) que não é atribuído apenas a condições de carga anormais. É crucial diferenciar (a) a variante sarcomérica, responsável pela etiologia principal da HVE inexplicada (40-60%) de (b) outras formas de CMH (variantes de genes não sarcoméricos ou etiologia genética não elucidada) e (c) outras causas genéticas e não genéticas, coletivamente denominadas mimetizadores de CMH (genocópias ou fenocópias).^2,3^ O objetivo deste artigo é fornecer uma metodologia pragmática para discriminar as diversas condições que resultam em HVE. Essa diferenciação considera diversos fatores, incluindo o perfil clínico do paciente, histórico familiar, atributos do eletrocardiograma (ECG), perfil laboratorial, características da ecocardiografia (ECO) e da ressonância magnética cardíaca (RMC) e, em casos selecionados, estudo genético e até biópsia endomiocárdica. O uso racional e abrangente da multimodalidade de imagem cardiovascular é de extrema importância para determinar um diagnóstico específico, fornecendo pistas para preencher lacunas. Na maioria das vezes, os exames de imagem são a peça que falta para resolver esse quebra-cabeça desafiador.

### Apresentação clínica

Para auxiliar no diagnóstico diferencial da HVE, o clínico-geral deve considerar uma abordagem focada, levando em conta a idade na primeira apresentação, os sintomas, o histórico pessoal e familiar e marcadores clínicos específicos no exame físico (“redflags”) (Tabela 1). A principal etapa investigativa em adultos que apresentam HVE envolve a triagem de etiologias frequentes, principalmente condições de sobrecarga de pressão, como HA e EA crônicas, ou adaptações fisiológicas associadas ao treinamento físico de alta performance (“coração do atleta”). Indivíduos com HVE podem ser assintomáticos ou apresentar sintomas inespecíficos, como dispneia ao esforço, fadiga, desconforto torácico, palpitações, síncope e/ou pré-síncope. A investigação de HVE é frequentemente precipitada por achados incidentais durante um ECG ou ECO, que geralmente são conduzidos para outros fins. Por outro lado, uma variedade de sintomas e sinais não cardíacos podem ocasionalmente ser indicativos de diagnósticos específicos.

**Tabela 1:** – Aspectos genéticos, epidemiológicos e clínicos de possível diagnóstico diferencial do fenótipo hipertrófico do ventrículo esquerdo

	Cardiomiopatia Hipertrófica	Doença de Fabry	Amiloidose cardíaca	Doença de Danon	Doença de Pompe	PRKAG2	RASopatias	Ataxia de Friedreich
Genética	• Herança autossômica dominante • Grande variedade de mutações genéticas, as mais prevalentes: MYH7 (cadeia pesada de miosina cardíaca beta) - 30-50%, MYBPC3 (proteína C de ligação à miosina) - 20-40%, TNNT2 (troponina T cardíaca) - 5-20%	• Herança ligada ao X • Mutação no gene GLA	• ATTR hereditário (mutante ou familiar): doença autossômica dominante com penetrância variável • Mutação do gene transtirretina (TTR)	• Herança ligada ao cromossomo X • Mutações no gene da membrana associada ao lisossomo 2 (LAMP2)	• doença de depósito lisossomal autossômica recessiva • Mutações no gene GAA	• Herança autossômica dominante • Mutações no gene PRKAG2	• Herança autossômica dominante • Mutações na via de sinalização RAS/MAPK	• Herança autossômica recessiva • Mutações no gene Frataxina (FXN)
Aspectos clínicos epidemiológicos	• HVE mais comum entre as causas genéticas • Pacientes jovens e mais velhos (ampla faixa etária) • História de síncope	• Opacidade da córnea • Angioceratoma • Hipoidrose • Albuminúria • Acroparestesia	• Adultos > 55 anos • Síndrome do túnel do carpo bilateral • Ruptura do tendão do bíceps • Polineuropatia periférica • Envolvimento sensorial, disfunção autonômica • Hematomas na pele	• Indivíduos do sexo masculino de 10 a 20 anos • Deficiência intelectual • Fraqueza muscular • Hepatomegalia • Mulheres com miocardiopatia quase exclusiva	• Crianças (ao nascer e primeiro ano de vida) / Adolescentes • Fraqueza muscular • Macroglossia • Retinite pigmentar	• Pacientes jovens (décadas I-IV) • Mialgia • Epilepsia • Hipertensão arterial de início precoce • Pré-excitação ventricular (pseudo Wolff-Parkinson-White)	• Crianças/adolescentes e pacientes jovens < 20 anos • Dismorfismo facial • Lentigos múltiplos • Pectus carinatum • Surdez • Cifose • Hipertelorismo	• > 15 anos • Ataxia simétrica da marcha • Cifoescoliose • Neuropatia sensorial • Disartria • Surdez • Vertigem

Em relação à idade de início, uma alta prevalência de doenças de armazenamento de glicogênio (por exemplo, doença de Pompe) e RASopatias (incluindo síndrome de Noonan) é observada como etiologia subjacente da HVE inexplicada em crianças e adolescentes. Já em adultos com mais de 55 anos, a amiloidose cardíaca (AC) pode ser encontrada com maior frequência, e estar ciente do possível diagnóstico desta doença tratável é muito importante.^4^ A CMH representa a etiologia mais comum para HVE entre as causas genéticas em diversas faixas etárias, abrangendo desde pacientes jovens até idosos.^4^

A HVE grave observada no nascimento ou durante o primeiro ano de vida, juntamente com fraqueza muscular, macroglossia e retinite pigmentar, deve levantar suspeita clínica de doença de Pompe. Em indivíduos do sexo masculino com idade entre 10 e 20 anos que apresentam HVE importante, deficiência intelectual, fraqueza muscular e pré-excitação ventricular, é necessária uma avaliação para doença de Danon. Da mesma forma, até os 20 anos, a presença de dismorfismo facial, lesões lentiginosas multiplas, pectus carinatum, surdez, cifose e hipertelorismo deve alertar os médicos sobre a possibilidade de RASopatias, como a síndrome de Noonan e a síndrome de Noonan com lentiginose multipla. Em indivíduos com mais de 15 anos, a ocorrência simultânea de sintomas neurológicos como ataxia, desequilíbrio e alterações na marcha pode indicar ataxia de Friedreich. Além disso, doenças mitocondriais, juntamente com HVE, frequentemente se manifestam com anormalidades sensoriais, bem como sintomas neurológicos e miopáticos. Em indivíduos com idade entre 30 e 40 anos, a doença de Fabry e a cardiomiopatia (CM) PRKAG2 devem ser incluídas no diagnóstico diferencial.^5^ A manifestação de sintomas gastrointestinais, dor neuropática, angioceratomas, hipoidrose, córnea verticilata, proteinúria, distúrbios de condução, ataque isquêmico transitório juvenil ou criptogênico, ou acidente vascular cerebral e perda auditiva, juntamente com histórico de transmissão hereditária ligada ao cromossomo X, levam à investigação da doença de Fabry.^6^ Em pacientes com mais de 55-60 anos, AC (transtirretina de cadeia leve ou de tipo selvagem) deve ser considerada, especialmente na presença de indicadores clínicos, como síndrome do túnel do carpo, ruptura espontânea do tendão do bíceps (sinal de Popeye), dor nas costas (indicativa de estenose espinhal), polineuropatia (manifestada como dor neuropática, dificuldades de locomoção ou quedas frequentes), intolerância a medicamentos anti-hipertensivos ou para insuficiência cardíaca devido à hipotensão postural, baixa voltagem do QRS desproporcional à massa do VE no ECO, insuficiência cardíaca com fração de ejeção preservada (ICFEp) e bradiarritmia.^7^

Quando há suspeita de etiologia genética, é fundamental realizar uma investigação detalhada do histórico familiar de três gerações, com foco no diagnóstico de CMH, presença de morte súbita, arritmia, implante de dispositivo intracardíaco e relatos de acidente vascular cerebral precoce.^5^ Mutações HCM e PRKAG2 são normalmente associadas à herança autossômica dominante. Um padrão ligado ao X deve sugerir a possibilidade da doença de Fabry ou Danon, ao passo que um padrão autossômico recessivo sugere ataxia de Friedreich^8^ (Tabela 1). Durante o exame físico, a presença de sinais indicativos de obstrução dinâmica da via de saída do VE (VSVE), como sopro sistólico que aumenta na posição ortostática ou pulso bífido, pode sugerir obstrução intraventricular causada por CMH. Um histórico de implantação de marcapasso, vários membros familiares afetados e a presença da síndrome de Wolff-Parkinson-White fundamentam o diagnóstico de PRKAG2, com a doença de Fabry como um possível diagnóstico alternativo.^9^

Dados clínicos isoladamente, embora informativos, são insuficientes para diferenciar a etiologia da HVE, e exames diagnósticos adicionais são essenciais para confirmar a causa subjacente. No entanto, um julgamento clínico amplo e uma avaliação personalizada de cada paciente são fundamentais para orientar a seleção criteriosa de metodologias diagnósticas apropriadas.

Em suma, com base nos critérios acima mencionados, três principais achados devem levantar suspeitas e iniciar a investigação de mimetizadores de CMH:

• Idade em que ocorre o início da HVE, seja no início ou em fase mais tardia da vida.
• A presença de manifestações extracardíacas.
• Padrões de herança que não são consistentes com a transmissão autossômica dominante.

### Eletrocardiografia

**Figura 2:**
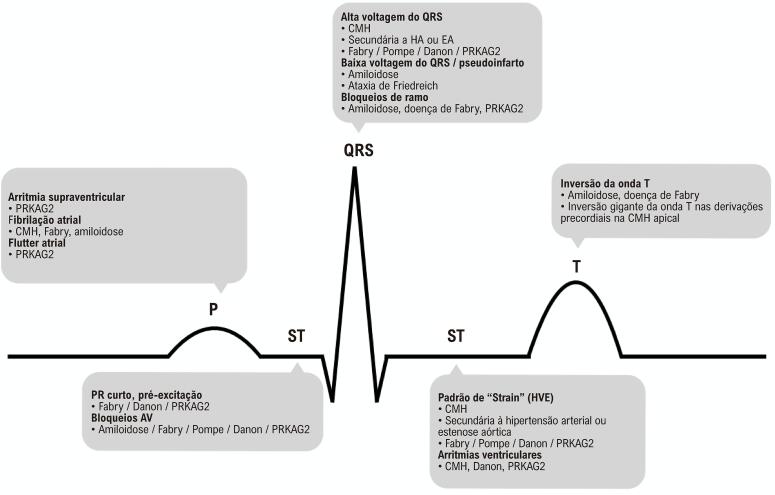
– Pistas eletrocardiográficas para o diagnóstico diferencial de HVE. CMH: miocardiopatia hipertrófica; AV: atrioventricular; HA: hipertensão arterial; EA: estenose aórtica; HVE: hipertrofia ventricular esquerda.

Anomalias eletrocardiográficas podem se manifestar anos antes do desenvolvimento de um fenótipo hipertrófico. Embora as alterações no ECG sejam geralmente inespecíficas, elas podem fornecer dicas importantes para o diagnóstico, especialmente quando interpretadas em conjunto com outros achados clínicos e laboratoriais e correlacionadas com multimodalidade de imagem (Figura 2). A CMH pode apresentar diversos padrões, incluindo “strain” do VE , com e anormalidades nas ondas ST e T, embora, em alguns casos, o ECG possa ser normal.^10^ Ondas T negativas profundas nas derivações precordiais podem sugerir CMH apical. Um padrão extremo de HVE sugere cardiomiopatias de Danon, Pompe e PRKAG2. Baixa voltagem do QRS (absoluta ou relativa, por exemplo, voltagem do QRS desproporcional à espessura da parede do VE), bloqueio atrioventricular e um padrão de pseudoinfarto são características da AC. Um curto intervalo PR/pré-excitação ventricular (principalmente em pacientes mais jovens) e bloqueios atrioventriculares (em pacientes adultos) são observados nas doenças de Fabry, Danon e PRKAG2. Bloqueios bifasciculares também podem indicar doença de Fabry como possível diagnóstico.^11^

Pacientes com HVE podem apresentar diversos tipos de arritmias, desde batimentos atriais e/ou ventriculares prematuros assintomáticos até arritmias ventriculares (AVs) possivelmente fatais. A fibrilação atrial (FA) é uma complicação comum na progressão clínica da CMH, doença de Fabry e amiloidose. Da mesma forma, CMH sarcomérica, doença de Danon e cardiomiopatias PRKAG2 estão associadas a um risco de AVs possivelmente fatais.^8^

### Exames laboratoriais

No contexto de um fenótipo hipertrófico e suas manifestações clínicas, exames laboratoriais de rotina e direcionados podem fornecer indicações para diagnósticos específicos. Embora não sejam específicos, níveis desproporcionalmente altos de peptídeo natriurético cerebral N-terminal (NT-proBNP) e pequenas elevações na troponina sérica podem indicar um diagnóstico de amiloidose ou formas específicas de CMH sarcomérica. Níveis séricos de creatina quinase (CK) persistentemente altos podem indicar doença de Pompe, doenças neuromusculares ou coração de atleta. Disfunção hepática, caracterizada por níveis séricos elevados de transaminases hepáticas, pode ser observada em cardiomiopatias de Pompe, Danon e PRKAG2. Imunoglobulina de cadeia leve em ensaios de imunofixação sérica e urinária e uma proporção anormal de cadeias leves livres são consistentes com o diagnóstico de amiloidose de cadeia leve (AL).^7^ Para a doença de Fabry, o “dry spot test” é uma ferramenta de triagem útil em homens, nos quais o diagnóstico é estabelecido por meio da avaliação da atividade da alfa-galactosidase A (α-GalA) e das medições de liso-Gb3. Em pacientes do sexo feminino, geralmente são necessários exames genéticos para confirmar o diagnóstico.

### Ecocardiograma

O ECO desempenha um papel fundamental no diagnóstico e tratamento da HVE, não apenas devido à sua ampla disponibilidade, natureza não invasiva e relativa acessibilidade, mas principalmente devido às informações abrangentes que fornece. Essas informações incluem visualização anatômica de estruturas (fenótipo de HVE, espessura das paredes do VE e distribuição geométrica de hipertrofia), avaliação da função ventricular esquerda e direita (VD) e avaliações hemodinâmicas (como pressão diastólica final do VE, pressão sistólica da artéria pulmonar, volume sistólico e colapsibilidade da veia cava). O ECO também é valioso para identificar obstruções fixas, como EA, ou obstruções dinâmicas do VE, como CMH obstrutiva.^12^

**Figura 3:**
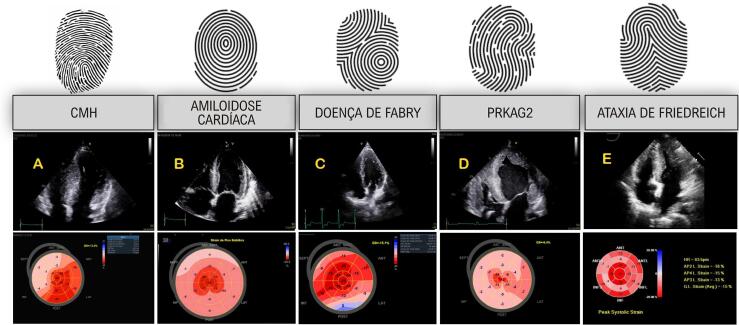
– Padrões de deformação longitudinal no fenótipo hipertrófico do ventrículo esquerdo (VE) (display paramétrico em “bulls-eye”). A) Cardiomiopatia hipertrófica (CMH) mostrando alterações de deformação regionais conforme a distribuição da hipertrofia (neste caso uma CMH assimétrica septal), B)- Amiloidose cardíaca, com padrão “cherry on top”, um “redflag” ecocardiográfico para seu diagnóstico, C) Doença de Fabry, com alteração típica de deformação na parede ântero-lateral basal, D) Cardiomiopatia PRKAG2, com hipertrofia biventricular maciça neste caso, com alteração global acentuada na GLS mostrando padrão difuso, E) Ataxia de Friedreich, com hipertrofia concêntrica do VE, mostrando alteração de deformação principalmente nos segmentos basal e medial, destacando que o padrão “cherry on top” (apical sparing) relativo não é específico para o diagnóstico de AC (este caso: cortesia - Dr. Thiago Santos Rosa - Brasil).

Avanços recentes em técnicas ecocardiográficas, particularmente na análise de deformação miocárdica, aumentaram nossa compreensão da fisiopatologia, da mecânica miocárdica e da função miocárdica além da fração de ejeção. A ecocardiografia com “speckle tracking”(STE) surgiu como uma ferramenta sensível para a detecção precoce de doenças miocárdicas, uma vez que o *strain* longitudinal global (GLS) geralmente se deteriora antes que a fração de ejeção do VE (FEVE) diminua em vários contextos clínicos, com a vantagem de ser menos dependente da carga. É importante para o diagnóstico de doenças cardíacas subclínicas em parentes genótipo-positivos de pacientes com CMH, ataxia de Friedreich e AC, bem como para o monitoramento de pacientes com doenças metabólicas, infiltrativas ou de armazenamento miocárdico. O STE auxilia na caracterização dos padrões de envolvimento miocárdico, atuando como uma espécie de “impressão digital” visual, servindo como uma “ferramenta de caracterização tecidual” baseada no ECO (Figura 3).^13^ Há uma forte correlação entre os valores de GLS e o realce tardio de gadolínio (LGE) na ressonância magnética cardíaca (RMC), apontando para fibrose miocárdica e maior risco de mortalidade e AVs malignas em pacientes com CMH.^14,15^ Este parâmetro também tem valor prognóstico em doenças infiltrativas como AC.^16^

O *“myocardial work”* foi recentemente descrito como uma nova ferramenta ecocardiográfica promissora para avaliação da mecânica miocárdica, incorporando a pós-carga (pressão arterial) como estimativa da pressão do VE e utilizando a deformação longitudinal (LS) para criar um loop “pressão x deformação”, gerado por um software específico.^17^ O valor adicional desta técnica em relação aos parâmetros ecocardiográficos convencionais para avaliação de cardiomiopatias ainda precisa ser comprovado em estudos maiores, mas alguns dados demonstram valor prognóstico para avaliação de CMH^18^ e AC,^19^ por exemplo.

Embora ainda não esteja universalmente disponível, a ecocardiografia tridimensional (3DE) tornou-se uma ferramenta valiosa para avaliar doenças miocárdicas, especialmente medições volumétricas diretas de câmaras cardíacas. Fornece valores precisos de FE e massa do VE, correlacionando-se bem com a RMC considerada padrão ouro.^20^ O 3DE também permite a medição tridimensional da deformação miocárdica, menos afetada por limitações técnicas, como movimento fora do plano, e permite a avaliação simultânea de todo o VE, o que é útil para análise de sincronização.

O ECO com contraste que utiliza agentes de realce ultrassonográficos é importante para a delimitação da borda do VE, especialmente em pacientes com janelas acústicas subótimas. Aumenta a sensibilidade na detecção de condições como CMH apical, aneurismas apicais e na diferenciação de trombos intracavitários e em outras estruturas, como tendões ou trabeculações.^21^

### Ressonância magnética cardiovascular

A RMC assumiu um papel inquestionável na avaliação da HVE, principalmente devido à avaliação da morfologia cardíaca, função e caracterização tecidual.^8^ Embora a RMC possa fornecer pistas essenciais para o diagnóstico final em entidades com anormalidades extremas na caracterização do tecido, como AC ou doença de Fabry, a grande sobreposição de achados de imagem em muitas entidades torna necessária uma integração abrangente dos achados de imagem no contexto clínico. Nenhum achado de imagem deve ser interpretado isoladamente, sem consideração da história clínica, dados eletrocardiográficos e história familiar.^22^

Além da avaliação anatômica e funcional do VE, a principal vantagem da RMC em relação ao ECO é a possibilidade de caracterização tecidual. Técnicas de mapeamento paramétrico que medem os tempos de relaxamento T1 e T2 têm sido cada vez mais incorporadas aos protocolos de aquisição, permitindo a avaliação quantitativa de componentes intracelulares e extracelulares.^23^Imagens com LGE podem identificar fibrose de substituição e possuem valor prognóstico bem estabelecido, embora sejam menos sensíveis para detectar deposição difusa de colágeno intersticial do que as técnicas de mapeamento T1.^24^

### Diagnóstico de hipertrofia ventricular esquerda

Embora as diretrizes clínicas estabeleçam valores de referência normais para a massa do VE de acordo com o gênero,^25^ desafios técnicos podem causar variabilidade e dificultar a medição ecocardiográfica da espessura da parede do VE. Janela acústica subótima, medições incorretas (oblíquas ou encurtadas, uso de janela apical – baixa resolução lateral) e inclusão de estruturas de confusão podem afetar a precisão. Algumas estruturas, como uma banda moderadora proeminente do VD, aparelho subvalvar tricuspídeo, crista supraventricular ou falsos tendões fibromusculares do VE inseridos no septo interventricular, podem ser erroneamente interpretadas como parte do septo, superestimando sua espessura. Em pacientes pediátricos, os escores Z, que representam o número de desvios padrão dos valores médios, são usados como padrões de referência. Esses escores ajustam a massa do VE e a espessura da parede conforme a idade e o tamanho corporal da criança, proporcionando uma avaliação mais personalizada nessa população.^3^ Os limiares para portadores de variantes genéticas patogênicas podem ser menores^12^ e algumas apresentações podem causar confusão e diagnósticos incorretos, como fenótipos tardios e já dilatados.^26^ Para garantir um diagnóstico preciso, é essencial correlacionar essas medidas com o histórico clínico, a presença de outras doenças cardíacas estruturais associadas, os valores de GLS e a função diastólica. Em certos casos, uma RMC pode ser necessária para confirmar o diagnóstico.

### Cardiomiopatia hipertrófica

**Figura 4:**
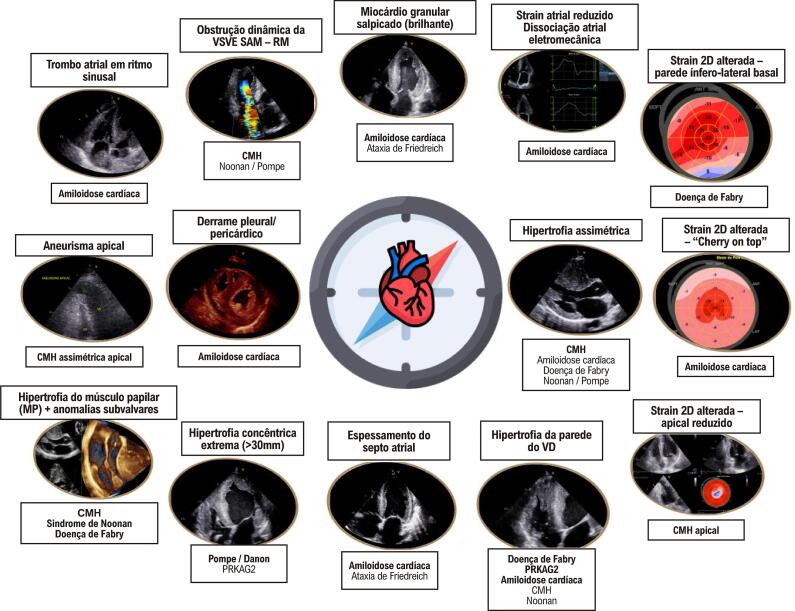
– Pistas ecocardiográficas para o diagnóstico diferencial do fenótipo hipertrófico do ventrículo esquerdo. CMH: cardiomiopatia hipertrófica; SAM: movimento anterior sistólico da valva mitral; RM: regurgitação mitral; VD: ventrículo direito.

A CMH é definida por um aumento da espessura da parede do VE ≥ 15 mm ou ≥ 13 mm (em indivíduos com genótipo positivo ou parentes de pacientes com CMH), na ausência de condições que justifiquem HVE secundária, como HA grave, EA ou coarctação da aorta, e excluindo quaisquer doenças sistêmicas infiltrativas.^27^ É crucial reconhecer que a CMH não é meramente uma doença miocárdica. Outras características que justificam o diagnóstico incluem anormalidades da VM e do aparelho subvalvar, como alongamento do folheto mitral, hipertrofia do músculo papilar, cordas da VM secundárias anormais e feixes musculares (Figura 4).^8,28,29^

A HVE septal assimétrica é o padrão mais clássico de CMH, mas outras expressões fenotípicas, como apical, concêntrica, de parede lateral, médio-ventricular, e apresentações menos típicas envolvendo qualquer segmento do VE, são comuns. Os critérios clássicos para diagnóstico de CMH, que são os mesmos utilizados no ECO e na RMC,^8^ foram recentemente questionados (“uma medida serve para todos”?). No futuro, provavelmente gênero, superfície corporal e raça serão considerados para a definição de novos limiares. Em especial, esses critérios podem não ser preenchidos na variante apical, caracterizada pela perda ou reversão do estreitamento miocárdico apical usual. Por exemplo, novos valores de corte e critérios diagnósticos foram recentemente sugeridos para a detecção de CMH apical, sendo o limite superior do normal apical de 11 mm ou 5,6 mm/m^2^.^22,30^

A hipertrofia do VD também é frequente em pacientes com CMH, sendo encontrada em 30-44% dos casos, geralmente junto da HVE.^31,32^ A obstrução dinâmica do VD pode ocorrer, seja intraventricular ou na via de saída do VD.^33^ Os índices ecocardiográficos convencionais da função sistólica do VD, como excursão sistólica do plano anular tricúspide (TAPSE), fração de variação de área (FAC) e velocidades do Doppler tecidual, são tipicamente normais, mas a disfunção sistólica subclínica pode ser identificada por meio de alterações na LS do VD.^34,35^

O ECO também desempenhou um papel importante na avaliação de risco e estratificação de pacientes com CMH,^36^ sendo que estudos demonstraram maior mortalidade em pacientes com CMH com espessura do septo do VE ≥ 30 mm, aneurisma apical ou disfunção do VE (FEVE < 50%).^3,37^ GLS 2D do VE correlaciona-se fortemente com fibrose em pacientes com CMH. Os valores absolutos de GLS e dispersão mecânica têm uma boa correlação com a porcentagem de LGE e são preditores independentes de AVs.^15^ No estudo de Reant et al.,^14^ valores absolutos de GLS < 15,4% foram associados à insuficiência cardíaca, morte e internações hospitalares em uma coorte de pacientes com CMH. O gráfico paramétrico da LS 2D derivado do STE2D (*“bulls-eye”*) pode oferecer uma visão geral intuitiva da deformação miocárdica global e regional do VE na CMH e é caracterizado por valores de deformação segmentar severamente reduzidos nas paredes mais hipertrofiadas, geralmente mais pronunciados do que outras etiologias, como hipertensão ou HVE secundária à EA.^38^

**Figura 5:**
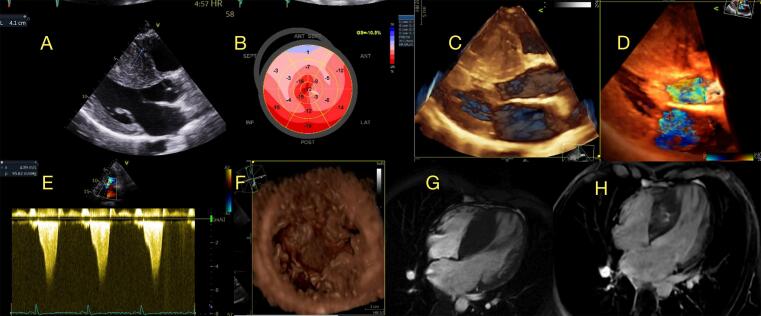
– Exemplo de paciente com Cardiomiopatia Hipertrófica Obstrutiva. A) espessura do septo: 4,1 cm, B) Padrão de deformação longitudinal “bulls-eye” mostrando alteração regional principalmente nos segmentos septais e, C) Aquisição de eco transtorácico (ETT) 3D, imagens 3D renderizadas longitudinais mostrando movimento sistólico anterior (SAM) da valva mitral na sístole (*), D) Aquisição de ETT 3D, imagens 3D coloridas renderizadas (visão longitudinal) mostrando regurgitação mitral secundária a SAM (*), E) Doppler contínuo mostrando gradiente de pico tardio em repouso na via de saída do ventrículo esquerdo de 96 mmHg, padrão clássico em forma de adaga, F) Aquisição de ETT 3D, imagens 3D renderizadas (visão de eixo curto) mostrando anomalia dos músculos papilares com quatro cabeças (*) e posicionados anteriormente, G) RMC mostrando hipertrofia septal maciça, e H) imagens com realce tardio com gadolínio mostrando fibrose septal com padrão de parede média.

A avaliação hemodinâmica para identificar pacientes com obstrução da VSVE é crucial para o tratamento de pacientes com CMH. Aproximadamente um terço dos pacientes com CMH apresentam obstrução da VSVE em repouso (> 30 mmHg), sendo que o outro terço apresenta obstrução latente, revelada por meio de manobras provocativas à beira do leito (Valsalva, ficar em pé, agachar-elevar, inalação de nitrito de amila) ou ecocardiografia de exercício.^39^A regurgitação mitral é um achado comum em pacientes com CMH, especialmente em pacientes com movimento mitral anterior sistólico (SAM), e pode ser um determinante importante dos sintomas. É muito importante destacar que o SAM não é resultado unicamente de hipertrofia assimétrica septal (altas velocidades da VSVE e efeito Venturi) e, de fato, anormalidades primárias do aparelho da VM, como hipertrofia e deslocamento anterior dos músculos papilares, alongamento dos folhetos e alteração na inserção cordal, podem ter um grande papel na obstrução da VSVE (Figura 5).^28,40^

A CMH geralmente se apresenta com FEVE normal ou aumentada, o fenótipo clássico da ICFEp, associada à disfunção diastólica em vários graus. Alguns pacientes podem apresentar aneurismas apicais e disfunção progressiva do VE, o que pode levar à doença cardíaca terminal ou à variante CMH *“burned-out”*, associada a um pior prognóstico.^41,42^

A RMC também permite uma caracterização anatômica detalhada em relação ao padrão de HVE, variações no aparelho da VM e suas contribuições para a obstrução da VSVE.^43^Considerando esta avaliação detalhada, a RMC desempenha um papel importante no planejamento de terapias de redução septal.^44^

Em relação à caracterização tecidual, T1 nativo e VEC correlacionam-se com fibrose difusa, elevada mesmo em áreas sem LGE.^45,46^ A fibrose de substituição típica com padrão de parede média, mais frequente em áreas hipertróficas, é bem descrita com LGE, com valor prognóstico bem reconhecido.^47^Com avanços recentes, *“diffusion tensor acquisition”* permite estudar a desorganização dos miócitos, um marcador prematuro da doença,^48^ e a RMC de perfusão sob estresse permite o estudo da disfunção microvascular.^49,50^

### Mimetizadores de CMH

#### Amiloidose cardíaca

A AC é uma CM infiltrativa causada pela deposição extracelular de fibrilas amiloides, com fenótipo clássico de HVE (“pseudo-hipertrofia”) e ICFEp. O ECO, particularmente nos estágios iniciais,^51^ carece especificidade para distinguir precisamente doenças cardíacas infiltrativas ou hipertróficas amiloides de não amiloides, reforçando a necessidade de correlação com outros (“redflags”) clínicos e complementação com outras modalidades de imagem. Os achados clássicos do ECO podem ser observados apenas em estágios avançados de infiltração amiloide, com aumento biatrial, espessamento das válvulas e do septo interatrial, derrame pleural e pericárdico, baixas velocidades miocárdicas e hipertrofia biventricular com aparência brilhante. Geralmente, esses pacientes apresentam FEVE preservada e disfunção diastólica acentuada com aumento das pressões de enchimento do VE (disfunção diastólica tipo II-III), embora a FEVE reduzida seja um achado frequente na doença em estágio avançado.^52,53^ É importante observar que pacientes com AC podem apresentar redução do débito sistólico mesmo antes de uma redução na FEVE, causada por diferentes fatores, incluindo alteração na deformação miocárdica, desempenho diastólico do VE prejudicado, disfunção mecânica atrial e volumes reduzidos do VE devido ao espessamento da parede.^54,55^

O ECO é um instrumento vital para o diagnóstico precoce de AC, particularmente em pacientes com fenótipo hipertrófico (definido como espessura da parede do VE ≥ 12 mm) combinado com outros “redflags” clínicos ou ecocardiográficos. Esses achados (Figura 4) devem levar o clínico a direcionar esses pacientes para um caminho investigativo especializado. Esse caminho normalmente inclui RMC, cintilografia com marcadores ósseos e quantificação de cadeias leves livres no soro, juntamente com testes de imunofixação no soro e na urina. Essas modalidades diagnósticas ajudam a confirmar o diagnóstico de AC, diferenciando os tipos de imunoglobulina de cadeia leve (AL) monoclonal dos tipos de transtirretina (ATTR) e avaliam o estadiamento da doença e o prognóstico.^56^

**Figura 6:**
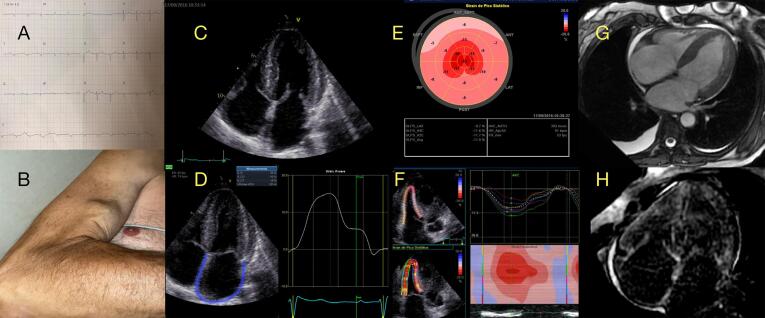
– Exemplo de paciente com Amiloidose Cardíaca. A) ECG com baixa voltagem nas derivações frontais, bloqueio atrioventricular de primeiro grau e padrão de “pseudoinfarto” nas derivações precordiais, B) um sinal clínico de ruptura do tendão do bíceps, C) 4 câmaras apicais mostrando aumento biatrial, espessamento do septo interatrial e das válvulas atrioventriculares, hipertrofia concêntrica do ventrículo esquerdo (VE), D) Strain atrial com componente de reservatório reduzido (+15%) geralmente causado por miopatia atrial e disfunção diastólica, E) Strain longitudinal (LS) do VE em formato de “bulls-eye” (exibição paramétrica) mostrando Strain regional alterada nos segmentos basal e medial, relativamente preservada nos segmentos apicais (“cherry on top”). F) LS do ventrículo direito (VD) reduzida (Strain da parede livre = -13%) mostrando infiltração de amiloide na parede do VD, G) RMC mostrando espessamento da parede biventricular, septo interatrial, aumento biatrial, H) RMC com realce tardio de gadolínio mostrando padrão global subendocárdico, com cinética de gadolínio alterada.

A GLS é notavelmente prejudicada em pacientes com AC, demonstrando uma forte correlação com a extensão da carga amiloide, conforme demonstrado em estudos que comparam a GLS com o LGE e o volume extracelular (VEC) medido por RMC.^13,54^ Um padrão regional característico de deformação longitudinal preservada nos segmentos apicais, formando um gradiente basal-apical ou um padrão de preservação apical relativa (RASp), foi identificado na AC (Figura 6). Existem muitas maneiras de identificar esse padrão por STE, usando diferentes fórmulas e critérios quantitativos ou mesmo considerando uma aparência visual de “cherry on top” um sinal visual qualitativo derivado da análise paramétrica do LS (“bulls eye”). O RASp demonstrou boa precisão na distinção de AC de outras causas de HVE e doenças miocárdicas.^13,57,58^Embora o RASp não seja específico para o diagnóstico de AC^59^ e possa ser encontrado em outras causas de HVE, ele pode ser usado como um valioso *redflag* ecocardiográfico para garantir investigação adicional em pacientes com histórico clínico compatível^60^ e também para determinar o prognóstico nesses pacientes.^61^ É importante enfatizar que o RASp pode ser observado em diferentes tipos de AC, incluindo AL, ATTR hereditário e amiloidose ATTR de tipo selvagem, e não é útil para distingui-los. Esse padrão pode não estar presente em uma proporção significativa de pacientes, porque nos estágios iniciais da doença apenas graus leves de infiltração amiloide em segmentos basais podem estar presentes. Por outro lado, um padrão difuso de envolvimento do miocárdio pode ocorrer na doença em estágio avançado, sem um gradiente significativo entre o ápice e a base do coração.^62^ Considerando a queda desproporcional e precoce da GLS e a FE relativamente preservada em pacientes com AC, a razão da FEVE dividida pela GLS apresentou boa acurácia para diferenciar a AC da CMH, com ponto de corte de 4,1.^63^

O strain miocárdico do VD é tipicamente prejudicado em pacientes com AC, o que pode ser uma característica diagnóstica útil para diferenciar a AC de outras causas de fenótipos hipertróficos. Curiosamente, um padrão de preservação apical relativa do VD, semelhante ao observado no VE, também foi identificado nesses pacientes. A identificação desse padrão do VD, juntamente com os achados do VE, aumenta a especificidade diagnóstica para AC, conforme citado em algumas publicações.^64,65^

**Figura 7:**
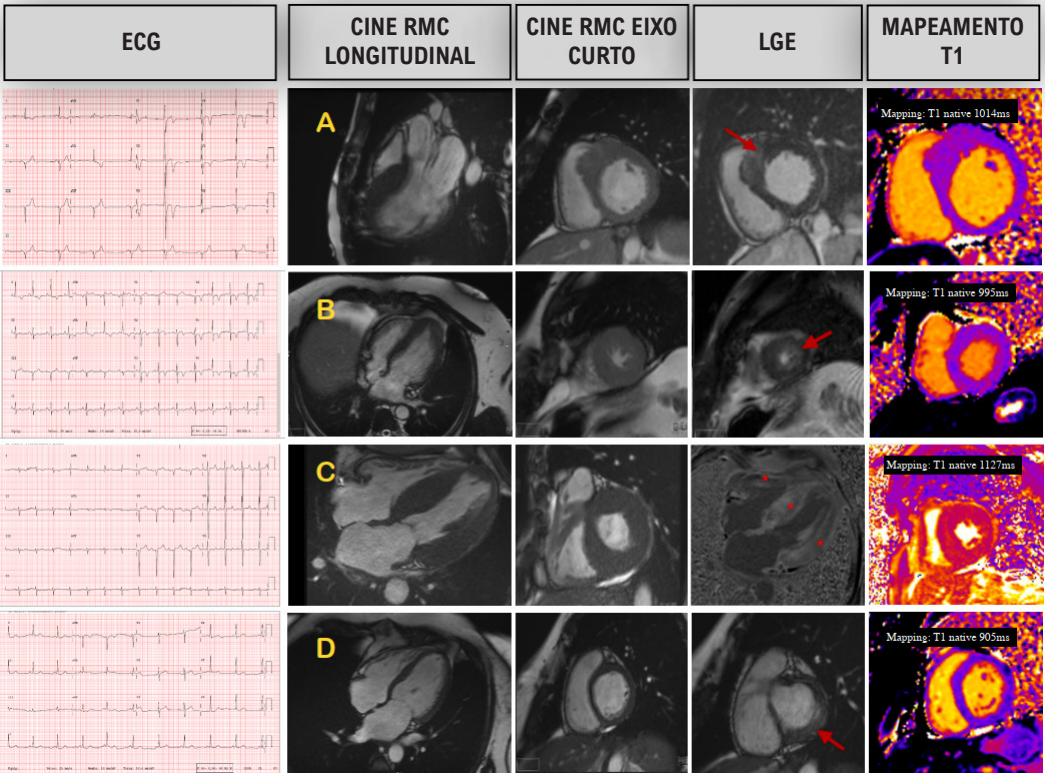
– Uso da ressonância magnética cardíaca (RMC) em pacientes com fenótipo hipertrófico do ventrículo esquerdo (VE), com imagens de ECG, cine RMC, realce tardio com gadolínio (LGE) e mapeamento de sequência T1. Em A) um paciente com cardiomiopatia hipertrófica septal assimétrica, em B) um paciente com cardiomiopatia hipertrófica apical, C) um paciente com amiloidose cardíaca (AC) ATTR, D) um paciente com doença de Fabry. As setas mostram áreas de fibrose e o (*) aponta para um padrão difuso típico na AC. Vale ressaltar que o mapeamento T1 mostra um aumento do T1 nativo no paciente com AC (C) e um tempo T1 reduzido em um paciente com doença de Fabry (D).

A RMC é particularmente útil para a AC, uma vez que os valores T1 são notavelmente extremos e o LGE tem cinética típica (Figura 7).^22^ O miocárdio amiloidótico tem uma avidez singular pelo gadolínio, levando a um “ponto nulo” miocárdico antes do pool sanguíneo do VE. Como contraste extracelular, o gadolínio se acumula na presença de expansão do espaço extracelular secundária à deposição de amiloide.^66^ O padrão do LGE é tipicamente global e subendocárdico, tornando-se transmural em estágios avançados.^67^Devido ao acúmulo extracelular de fibrilas amiloides, o VEC aumenta acentuadamente, frequentemente acima de 40%.^68^

Quando utilizada isoladamente, a RMC não permite uma distinção precisa entre amiloidose AL e ATTR, embora cada tipo tenham algumas características específicas: O LGE do VD fica aparente na maioria dos pacientes com amiloidose ATTR, mas apenas em cerca de 70% dos pacientes com amiloidose AL; a massa do VE e o VEC são maiores no tipo ATTR, enquanto T1 e T2 nativos são maiores na amiloidose AL secundária à toxicidade da cadeia leve nos cardiomiócitos.^67,68^Além disso, o cálculo do VEC no fígado e no baço pode identificar envolvimento sistêmico na AL, o que é muito raro na ATTR.^69^

A combinação de imagens multimodais e a observação de suas características podem fornecer pistas importantes para identificar possíveis diagnósticos diferenciais. Características da amiloidose na RMC com grau 0 ou 1 na cintilografia óssea apontam para o diagnóstico de amiloidose AL, ou raramente, variantes TTR, AApoAI e AApoAIV-amiloidose.^70^ Quando combinada com estudos de proteínas monoclonais normais, a presença de características na RMC mostra alta especificidade para o diagnóstico de AC ATTR.^71^

#### Doença de Fabry

A doença de Fabry é uma doença rara de armazenamento lisossomal causada por uma deficiência de α-GalA. A HVE é a principal manifestação cardíaca da doença de Fabry, sendo responsável por 0,9% dos casos de CMH.^72^ Nos homens, o acúmulo crônico de globotriaosilceramida é responsável por 2% da hipertrofia cardíaca total, mas seu armazenamento desencadeia a expressão da proteína sarcomérica via hipertrofia dos miócitos. Por outro lado, nas mulheres, a HVE consiste em hipertrofia equilibrada de esfingolipídeos e miócitos em proporção. Muitas vezes, é subdiagnosticada e pode levar a desfechos ruins se não for tratada. O envolvimento cardíaco é o fator prognóstico mais crucial na doença de Fabry e impacta significativamente a qualidade de vida.^6^ Alterações cardíacas na doença de Fabry podem ser sutis em pacientes jovens, mas eles geralmente desenvolvem ICFEp, arritmias e HVE mimetizando CMH mais tardiamente, geralmente após os 30 anos nos homens e os 40 anos nas mulheres. O diagnóstico precoce é vital, especialmente porque a terapia de reposição enzimática está disponível e pode limitar a progressão da doença.^73^

As características ecocardiográficas típicas da doença de Fabry são HVE concêntrica com FE preservada e hipertrofia desproporcional dos músculos papilares (Figura 4). Entretanto, alguns pacientes podem apresentar HVE assimétrica e até mesmo obstrução dinâmica da VSVE, levando ao diagnóstico incorreto de CMH. Pode ocorrer dilatação da raiz aórtica e espessamento das válvulas mitral e aórtica, geralmente sem disfunção significativa. A hipertrofia do VD com função sistólica preservada é comum em pacientes com doença de Fabry e HVE, e esses pacientes geralmente apresentam melhor função sistólica em comparação aos pacientes com AC com níveis semelhantes de espessamento da parede do VD.^74^ A GLS é significativamente reduzida em pacientes com doença de Fabry evidente, e as alterações de deformação regional são frequentemente mais pronunciadas na parede ínfero-lateral basal, correlacionando-se com LGE nessa região na RMC. Para portadores de variantes patogênicas do gene GLA, GLS facilita a detecção precoce do envolvimento cardíaco, independentemente da HVE.^75^

A RMC mostra T1 nativo baixo,^76^ o que é característico, refletindo a deposição de esfingolipídeos. Uma vez que o armazenamento é principalmente um fenômeno intracelular, o espaço extracelular é poupado pelo acúmulo, resultando em um VEC normal medido pelo contraste pré e pós T1.^76^ De fato, a RMC pode retratar as três fases da história natural da doença. Na fase de acumulação inicial, um T1 nativo baixo é observado na ausência de HVE. A progressão da doença é documentada pelo aparecimento de hipertrofia, inflamação e LGE principalmente na parede ínfero-lateral basal. Por fim, na presença de LGE extenso, há uma pseudonormalização do T1^76^ nativo.

#### Síndrome de Noonan

A síndrome de Noonan é uma doença genética autossômica dominante, parte de um grupo conhecido como RASopatias, que afeta vários sistemas do corpo. Possui diversas características, incluindo anormalidades cardíacas congênitas, baixa estatura, pescoço alado, dismorfismo craniofacial, malformações esqueléticas, diátese hemorrágica, hipertelorismo e deficiência intelectual leve. Mutações que causam a síndrome de Noonan afetam genes que codificam proteínas da via RAS-MAPK (proteína quinase ativada por mitógeno). Isso leva à desregulação de processos celulares críticos, incluindo proliferação, diferenciação, sobrevivência e metabolismo, característicos das RASopatias.

Em mais de 80% dos pacientes com síndrome de Noonan, são observadas anormalidades cardíacas, com estenose pulmonar prevalente em aproximadamente 50% dos casos e CMH ocorrendo em 25%.^77^Além disso, a síndrome de Noonan está associada a um amplo espectro de outras malformações cardíacas.^78^A HVE geralmente se manifesta de forma precoce e é normalmente diagnosticada nos primeiros seis meses de vida.^79^A HVE observada na síndrome de Noonan pode se apresentar como concêntrica ou assimétrica, às vezes acompanhada de obstrução dinâmica da VSVE. Anomalias da VM e complicações subvalvares, como SAM, inserção anômala da VM levando à obstrução subaórtica e degeneração mixomatosa, resultando em prolapso da válvula, são comumente observadas em pacientes com síndrome de Noonan e CMH.^80^ A presença de CMH na síndrome de Noonan influencia significativamente com a evolução dos pacientes, correlacionando-se com aumento da morbidade e mortalidade.^81^ A progressão da HVE é variável; em alguns casos, pode surgir mais tarde na infância e progredir lentamente, permanecer estável por vários anos ou evoluir rapidamente durante a infância. Em um subconjunto de pacientes, representando 17% de uma coorte de 46 indivíduos acompanhados por sete anos, foram observadas regressão e estabilização da HVE.^82^

#### Doença de Pompe

A doença de Pompe é classificada como uma doença de depósito lisossomal autossômica recessiva de incidência rara, decorrente de mutações no gene da α-glicosidase ácida. Essa alteração genética resulta em um acúmulo de glicogênio lisossomal em vários tecidos, principalmente no miocárdio, no sistema respiratório e nos músculos esqueléticos. O início da doença de Pompe varia, com um possível diagnóstico ao nascimento, na infância ou na idade adulta. A forma clássica da doença, observada predominantemente em lactentes, é caracterizada por rápida progressão e geralmente se apresenta com CMH, muitas vezes com prognóstico desfavorável. Nesses casos, bebês não tratados frequentemente sucumbem à insuficiência cardiorrespiratória no primeiro ano de vida. Fenotipicamente, a doença de Pompe é marcada por HVE, predominantemente com espessamento septal assimétrico, embora também seja observada hipertrofia concêntrica envolvendo as paredes septal e livre do VE e do VD. Na RMC, o LGE é raro e pode ser visto no subendocárdio das paredes lateral e anterior. A hipertrofia septal grave leva, geralmente, à SAM e obstrução da VSVE, exacerbando os sintomas clínicos. Essas patologias podem evoluir para disfunção diastólica e sistólica, culminando em IC. Notavelmente, observou-se que a terapia de reposição enzimática induz a regressão rápida da HVE e melhora a função ventricular sistólica, conforme avaliado pela análise de deformação miocárdica.^83^

#### Cardiomiopatia PRKAG2

**Figura 8:**
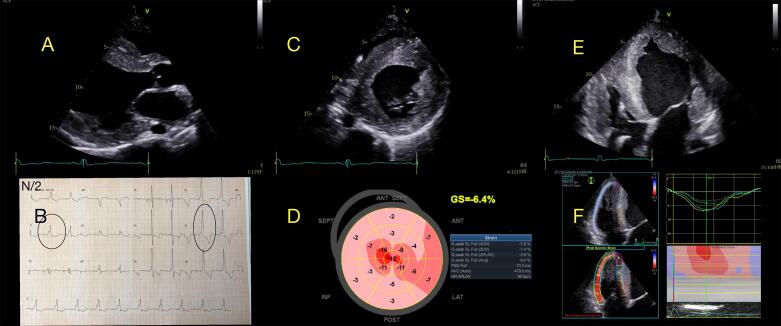
– Exemplo de um paciente jovem (homem de 25 anos) com cardiomiopatia PRKAG2. Em A) visão paraesternal do eixo longo mostrando um ventrículo esquerdo (VE) remodelado com hipertrofia. Em B) ECG mostrando ondas delta, intervalo PR curto (síndrome de Wolff-Parkinson-White) e padrão de hipertrofia do VE. Em C) visão de eixo curto representando hipertrofia concêntrica do VE. Em D) strain longitudinal (LS) do VE, Bulls-eye (display paramétrico) mostrando alteração difusa da deformação miocárdica. E) hipertrofia concêntrica biventricular e um DCI nas cavidades direitas. F) Strain longitudinal do ventrículo direito alterado (strain médio de parede livre = -9,33%).

A CM PRKAG2, uma doença autossômica dominante de armazenamento de glicogênio que afeta principalmente o músculo cardíaco e o sistema de condução, apresenta um perfil clínico e prognóstico únicos. A CM PRKAG2 é caracterizado por HVE, síndrome de Wolff-Parkinson-White e doença progressiva do sistema de condução.^84^A CMH em pacientes com mutações no gene PRKAG2 geralmente surge na adolescência ou na idade adulta, com poucos casos relatados na infância. A CM PRKAG2 está associada a piores desfechos em comparação à CMH sarcomérica, com pacientes potencialmente apresentando insuficiência cardíaca precoce e morte súbita.^85^ O fenótipo ecocardiográfico frequentemente mostra HVE concêntrica, FEVE preservada, disfunção diastólica e, menos comumente, hipertrofia do VD (Figura 8).^86,87^ A obstrução da VSVE é rara nesses pacientes, comparando-se aos pacientes com CMH sarcomérica. Um estudo mostrou que pacientes com CM PRKAG2 podem ter GLS mais preservada, associada à bradicardia, embora tenham massa do VE e espessamento das paredes ventriculares semelhantes.^88^

#### Ataxia de Friedreich

A ataxia de Friedreich é uma doença degenerativa autossômica recessiva, que afeta o gene da frataxina, levando ao armazenamento de ferro mitocondrial e afetando o metabolismo da glicose, o sistema nervoso e o coração.^89^ A doença cardíaca geralmente se manifesta como fenótipo de CMH que pode evoluir para cardiomiopatia dilatada, a causa mais importante de morte.^90^O envolvimento miocárdico pode ser detectado subclinicamente antes do desenvolvimento de HVE ou redução da FEVE, seja por STE^91^ ou RMC.^92^

As características ecocardiográficas típicas são HVE concêntrica sem obstrução da VSVE ou médio-ventricular (embora também possa ocorrer hipertrofia assimétrica), com disfunção do VE (FE reduzida) e insuficiência cardíaca em doença avançada^93^ com reserva de perfusão alterada.^8^ O aspecto morfológico da HVE nesses pacientes pode assemelhar-se à AC, mesmo com uma textura granular brilhante do miocárdio, mas geralmente não há aumento biatrial, derrame pericárdico ou disfunção diastólica grave em pacientes com ataxia de Friedreich.^94^

#### Doença de Danon

A doença de Danon é uma doença genética rara, dominante, ligada ao cromossomo X, que se manifesta com a tríade clínica de cardiomiopatia, miopatia esquelética e deficiência intelectual. É causada por mutações no gene da membrana associada ao lisossomo 2 (LAMP2). A cardiomiopatia de Danon é progressiva e geralmente se manifesta como fenótipo hipertrófico, com alterações do strain radial, circunferencial e longitudinal nos estágios iniciais, com fração de ejeção preservada (ICFEp). Com a progressão da fibrose, esses pacientes podem evoluir com declínio da fração de ejeção, piora dos sintomas e fenótipo dilatado, principalmente em pacientes do sexo masculino.^95^ A extensão e a gravidade da cardiomiopatia são os principais fatores prognósticos. A maioria dos pacientes é assintomática durante a infância, progredindo para um estágio sintomático durante a adolescência e culminando em insuficiência cardíaca fulminante e morte súbita na idade adulta.^96^ Os achados na RMC desempenham um papel fundamental no diagnóstico desta doença e geralmente incluem HVE acentuada, que pode ser concêntrica ou assimétrica, e padrões distintos de LGE que diferem de outras formas de cardiomiopatia hipertrófica. Geralmente, o LGE poupa o septo médio e exibe um gradiente da base para o ápice com envolvimento do ápice.^97,98^ Além disso, a RMC pode revelar T1 nativo e VEC elevados, sugerindo fibrose miocárdica.^98,99^

## Conclusão

A associação das diferentes peças do quebra-cabeça da HVE (histórico pessoal e familiar do paciente, apresentação clínica, achados do exame físico, características do ECG e informações obtidas em exames de multimodadlidade de imagem cardíaca) pode identificar *redflags* específicos que ajudam o clínico a diferenciar as causas de fenótipos hipertróficos.

Essa abordagem sistemática permite diagnósticos mais precisos e estratégias de tratamento personalizadas. No entanto, é importante observar que exames diagnósticos adicionais, como testes genéticos, teste de exercício cardiopulmonar e biópsia endomiocárdica, podem ser necessários para confirmar a etiologia subjacente. O julgamento clínico e a avaliação individualizada do paciente continuam sendo cruciais no processo de diagnóstico.
