# Supplementary material for: O Complexo Quebra-Cabeça do Fenótipo Hipertrófico: Uma Abordagem Prática para o Clínico
Source: Arq Bras Cardiol. 2025 Mar 18;122(1):e20240529. [Article in Portuguese] doi: 10.36660/abc.20240529 (PMC12013738; doi:10.36660/abc.20240529)
Supplement: Translation

# Translation: The Complex Puzzle of Hypertrophic Phenotype: A Practical Approach for the Clinician

**DOI:** 10.36660/abc.20240529i

**Authors:** Alex dos Santos Felix, Silvio Henrique Barberato, Marcelo Dantas Tavares de Melo, Sílvia Aguiar Rosa, Nuno Cardim

**Affiliations:** 1 Instituto Nacional de Cardiologia, Rio de Janeiro, RJ – Brazil; 2 DASA - Diagnósticos da América SA, Rio de Janeiro, RJ – Brazil; 3 Universidade do Estado do Rio de Janeiro, Rio de Janeiro, RJ – Brazil; 4 Complexo Hospitalar Américas - Vitória / Samaritano Barra, Rio de Janeiro, RJ – Brazil; 5 CardioEco Centro de Diagnóstico Cardiovascular, Curitiba, PR – Brazil; 6 Quanta Diagnóstico - Ecocardiografia, Curitiba, PR – Brazil; 7 Universidade Federal da Paraíba, João Pessoa, PB – Brazil; 8 Hospital of Santa Marta, Lisboa – Portugal; 9 NOVA Medical School, Lisboa – Portugal; 10 Hospital CUF Descobertas, Lisboa – Portugal

**Keywords:** Left Ventricular Hypertrophy, Diagnosis, Differential Diagnosis, Cardiac Imaging Techniques, Heart Function Tests

## Abstract

Left ventricular hypertrophy (LVH) represents a frequent observation in clinical practice. Nonetheless, the hypertrophic phenotype emerges as a common manifestation of diverse conditions, thereby presenting a diagnostic conundrum for clinicians. Differentiation among the etiologies of LVH is imperative for therapy decision-making, as different approaches must be implemented for distinct conditions, such as LVH secondary to loading changes, hypertrophic cardiomyopathy (HCM), or HCM mimics.

In some instances, an erroneous or late diagnosis may lead to a progression of the underlying disease with worsening functional capacity, high morbidity and mortality.

The rational use of cardiovascular multimodality imaging is of great importance when carried out in addition to a thorough clinical assessment and correlated with electrocardiographic findings, providing clues to fill the gaps, being, most of the time, the missing piece to solve this challenging puzzle.

An integrative approach is of paramount importance for the evaluation of these patients, as they are often followed by several specialties, with varied systemic manifestations. Although a multidisciplinary team is needed for an optimized follow-up of these patients, the most important player in this journey is the clinician, whose mission is to bring together all the red flags and coordinate all the data for an assertive diagnosis.

The objective of this review is to provide a pragmatic methodology, highlighting important clues for discriminating among the diverse conditions that result in LVH.

## Introduction

Left ventricular hypertrophy (LVH) consists of an increased LV wall thickness, representing a frequent observation in clinical practice. Nonetheless, the hypertrophic phenotype emerges as a common manifestation of diverse conditions, thereby presenting a diagnostic conundrum for clinicians.^1^ Differentiation among the etiologies of LVH (Figure 1) is imperative for devising precise management approaches. LVH frequently originates from secondary adaptive mechanisms, such as arterial hypertension (AH), aortic stenosis (AS), and athlete’s heart, or from a spectrum of other pathological states, encompassing both genetic and acquired diseases, that may concurrently exist. Hypertrophic cardiomyopathy (HCM) is characterized by LV wall thickening (≥ 15 mm anywhere in the left ventricle) that is not solely attributable to abnormal loading conditions. It is crucial to differentiate (a) the sarcomeric variant, which accounts for the principal etiology of unexplained LVH (40-60%), from (b) other HCM forms (variants of non-sarcomeric genes or unresolved genetic etiology) and (c) other genetic and non-genetic causes, collectively termed as HCM mimics (genocopies or phenocopies).^2,3^ The objective of this article is to provide a pragmatic methodology for discriminating among the diverse conditions that result in LVH. This differentiation considers an array of factors, including the patient’s clinical profile, family history, electrocardiogram (ECG) attributes, laboratory profile, echocardiography (ECHO), and cardiac magnetic resonance (CMR) features, and in selected cases, genetic study and even endomyocardial biopsy. A rational and comprehensive use of cardiovascular multimodality imaging is particularly important to point to a specific diagnosis, providing clues to fill the gaps, being most of the time, the missing piece to solve this challenging puzzle.

**Figure 1:** – Challenging puzzle of differential diagnosis in patients with left ventricular hypertrophic phenotype.

### Clinical presentation

To aid in the differential diagnosis of LVH, the clinician should keep in mind a focused approach, considering age at first presentation, symptoms, personal and family history, and specific clinical markers on physical examination (“red flags”) (Table 1). The primary investigative step in adults exhibiting LVH involves screening for frequent etiologies, notably pressure overload conditions, like chronic AH and AS, or physiological adaptations associated with athletic training. Individuals with LVH may be asymptomatic or exhibit nonspecific symptoms, such as exertional dyspnea, fatigue, chest discomfort, palpitations, syncope, and/or presyncope. The investigation of LVH is often precipitated by incidental findings identified during an ECG or ECHO, which are often conducted for other screening objectives. Conversely, a variety of noncardiac symptoms and signs may occasionally be indicative of specific diagnoses.

**Table 1:** – Genetics, epidemiological and clinical aspects of possible differential diagnosis of left ventricular hypertrophic phenotype

	Hypertrophic Cardiomyopathy	Fabry Disease	Cardiac Amyloidosis	Danon Disease	Pompe Disease	PRKAG2	RASopathies	Friedreich’s Ataxia
Genetics	• Autosomal dominant inheritance • Great variety of gene mutations, most prevalent: MYH7(cardiac myosin heavy chain beta) - 30-50%, MYBPC3 (Myosin binding protein C) -20-40%, TNNT2(Cardiac troponin T -5-20%	• X-linked inheritance • Mutation in GLA gene	• Hereditary ATTR (mutant or familiar): an autosomal-dominant disease with variable penetrance • Mutation in transthyretin (TTR) gene	• X-linked inheritance • Mutations in the lysosome-associated membrane 2(LAMP2) gene	• Autosomal recessive lysosomal storage disorder • Mutations in the GAA gene	• Autosomal dominant inheritance • Mutations in thePRKAG2 gene	• Autosomal dominant inheritance • Mutations in the RAS/MAPK signaling pathway	• Autosomal recessive inheritance • Mutations in the Frataxin gene (FXN)
Epidemiological Clinical aspects	• Most common LVH among genetic causes • Young and older patients (broad age spectrum) • History of syncope	• Corneal opacity • Angiokeratoma • Hypohidrosis • Albuminuria • Acroparesthesia	• Adults > 55 years old • Bilateral carpal tunnel syndrome • Biceps tendon rupture. • Peripheral polyneuropathy • Sensory involvement, autonomic dysfunction • Skin bruising	• Male individuals from 10-20 years old • Intellectual disability • Muscle weakness • Hepatomegaly • Women with almost exclusive cardiomyopathy	• Children (at birth and first year of life) / Adolescents • Muscle weakness • Macroglossia • Pigmentary retinitis	• Young patients(I-IV decade) • Myalgia • Epilepsia • Early onset arterial hypertension • Ventricular preexcitation(pseudo Wolff-Parkinson White)	• Children /Adolescents and young patients < 20 years old • Facial dysmorphism • Multiple lentigines • Pectus carinatum • Deafness • Kyphosis • Hypertelorism	• > 15 years old • Symmetric gait ataxia. • Kyphoscoliosis • Sensory neuropathy • Dysarthria • Deafness • Vertigo

Concerning the onset age, an elevated prevalence of glycogen storage diseases (e.g., Pompe disease) and RASopathies (including Noonan syndrome) is noted as underlying etiologies for unexplained LVH in children and adolescents. Conversely, in adults aged over 55 years, cardiac amyloidosis (CA) can be found at a higher frequency, and awareness of the possible diagnosis of this treatable disease is very important.^4^ HCM represents the most common etiology for LVH among genetic causes across a broad age spectrum, spanning from young to elderly patients.^4^

Severe LVH observed at birth or during the first year of life, coupled with muscle weakness, macroglossia, and pigmentary retinitis, should prompt clinical suspicion of Pompe disease. In male individuals aged between 10 and 20 years presenting with substantial LVH, intellectual disability, muscle weakness, and ventricular pre-excitation, an evaluation for Danon disease is warranted. Similarly, up to the age of 20, the presence of facial dysmorphism, multiple lentigines, pectus carinatum, deafness, kyphosis, and hypertelorism should alert clinicians to the possibility of RASopathies, such as Noonan syndrome and Noonan syndrome with multiple lentigines. In individuals over 15 years old, the co-occurrence of neurological symptoms like ataxia, imbalance, and alterations in gait may indicate Friedreich’s ataxia. Additionally, mitochondrial diseases, alongside LVH, often manifest with sensorial abnormalities as well as neurological and myopathic symptoms. In individuals aged between 30 and 40 years, Fabry disease and PRKAG2 cardiomyopathy (CM) should be included in the differential diagnosis.^5^ The manifestation of gastrointestinal symptoms, neuropathic pain, angiokeratomas, hypohidrosis, cornea verticillata, proteinuria, conduction disturbances, juvenile or cryptogenic transient ischemic attack or stroke, and hearing loss, coupled with a history of X-linked hereditary transmission, prompt to the investigation of Fabry disease.^6^ In patients aged over 55-60 years, CA (either light chain or wild-type transthyretin) should be considered, particularly in the presence of clinical indicators such as carpal tunnel syndrome, spontaneous biceps tendon rupture (Popeye’s sign), back pain (indicative of spinal stenosis), polyneuropathy (manifesting as neuropathic pain, ambulatory difficulties, or frequent falls), intolerance to antihypertensive or heart failure medications due to postural hypotension, disproportionate low voltage QRS in ECG to LV mass, heart failure with preserved ejection fraction (HFpEF), and bradyarrhythmia.^7^

When a genetic etiology is suspected, it is crucial to conduct a detailed inquiry into the three-generation family history, focusing on the diagnosis of HCM, presence of sudden death, arrhythmia, intracardiac device implantation, and reports of early stroke.^5^ HCM and PRKAG2 mutations are typically associated with autosomal dominant inheritance. An X-linked pattern should prompt consideration of Fabry or Danon disease, whereas an autosomal recessive pattern suggests Friedreich’s ataxia^8^(Table 1). During the physical examination, the presence of signs indicative of dynamic LV outflow tract obstruction (LVOT), such as a systolic murmur that increases in standing position or bifid pulse, may suggest intraventricular obstruction caused by HCM. A history of pacemaker implantation, numerous affected family members, and the presence of Wolff-Parkinson-White syndrome lend support to the diagnosis of PRKAG2, with Fabry disease as a potential alternative diagnosis.^9^

Clinical data alone, while informative, is insufficient for the differentiation of the etiology of LVH, and additional diagnostic tests are essential for confirming the underlying cause. Nonetheless, a comprehensive clinical judgment and a tailored assessment of each patient are critical in guiding the judicious selection of appropriate diagnostic methodologies.

In summary, based on the aforementioned criteria, three principal findings should prompt suspicion of, and initiate the investigation for HCM mimics:

• The age at which LVH onset occurs, whether early or late in life.
• The presence of extracardiac manifestations.
• Patterns of inheritance that are not consistent with autosomal dominant transmission.

### Electrocardiography

Electrocardiographic anomalies may manifest years before the development of a hypertrophic phenotype. While ECG changes are generally non-specific, they can provide diagnostic hints, especially when interpreted in conjunction with other clinical and laboratory findings and correlated with multimodality imaging (Figure 2). HCM may exhibit a wide range of patterns, including left ventricular strain and ST- and T-wave abnormalities, although, in some cases, the ECG can be normal.^10^ Deep negative T-waves in precordial leads may suggest apical HCM. An extreme LVH pattern is suggestive of Danon, Pompe, and PRKAG2 cardiomyopathies. Low QRS voltage (absolute or relative, e.g., disproportionate QRS voltage to LV wall thickness), atrioventricular block and a pseudoinfarction pattern are hallmarks of CA. A short PR interval/ventricular pre-excitation (notably in younger patients) and atrioventricular blocks (in adult patients) are observed in Fabry, Danon, and PRKAG2 diseases. Bifascicular blocks may also point to Fabry disease as a possible diagnosis.^11^

**Figure 2:** – Electrocardiographic clues for the differential diagnosis of LVH. HCM: hypertrophic cardiomyopathy; AV: atrioventricular; AH: arterial hypertension; AS: aortic stenosis; LVH: left ventricular hypertrophy.

Patients with LVH may experience a wide range of arrhythmias, from asymptomatic atrial and/or ventricular premature beats to life-threatening ventricular arrhythmias (VAs). Atrial fibrillation (AF) is a common complication in the clinical progression of HCM, Fabry disease, and amyloidosis. Similarly, sarcomeric HCM, Danon, and PRKAG2 cardiomyopathies are associated with a risk of life-threatening VAs.^8^

### Laboratory tests

Within the framework of a hypertrophic phenotype and its clinical manifestations, the application of routine and targeted laboratory investigations can provide indications for specific diagnoses. Although non-specific, disproportionately high levels of N-terminal pro-brain natriuretic peptide (NT-proBNP) and minor elevations in serum troponin may point to a diagnosis of amyloidosis or specific forms of sarcomeric HCM. Sustainedly high serum creatine kinase (CK) levels could indicate Pompe disease, neuromuscular diseases or athlete’s heart. Liver dysfunction, characterized by raised serum levels of hepatic transaminases, may be observed in Pompe, Danon, and PRKAG2 cardiomyopathies. The finding of light-chain immunoglobulin in serum and urine immunofixation assays and an abnormal free-light-chain ratio are consistent with a diagnosis of AL amyloidosis.^7^ For Fabry disease, the “dry spot test” is a useful screening tool in males, in whom the diagnosis is established through the assessment of alpha-galactosidase A (α-GalA) activity and lyso-Gb3 measurements. In female patients, genetic testing is typically required to confirm the diagnosis.

### Echocardiography

ECHO plays a pivotal role in the diagnosis and management of LVH, not only due to its broad availability, non-invasive nature, and relative affordability but predominantly because of the comprehensive information it provides. This includes anatomical visualization of structures (LVH phenotype, thickness of LV walls, and geometric distribution of hypertrophy), assessment of left and right ventricular (RV) function, and hemodynamic evaluations (such as LV end-diastolic pressure, pulmonary artery systolic pressure, stroke volume, and vena cava collapsibility). ECHO is also valuable for identifying fixed obstructions, like AS, or dynamic LV obstructions, like obstructive HCM.^12^

Recent advances in echocardiographic techniques, particularly in myocardial deformation analysis, have enhanced our understanding of pathophysiology, myocardial mechanics and myocardial function beyond ejection fraction. Speckle-tracking echocardiography (STE) has emerged as a sensitive tool for the early detection of myocardial disease, as global longitudinal strain (GLS) often deteriorates before LV ejection fraction (LVEF) decreases in various clinical contexts, with the advantage of being less load dependent. It is important for diagnosing subclinical cardiac diseases in genotype-positive relatives of patients with HCM, Friedreich’s ataxia, and CA, as well as for monitoring patients with metabolic, infiltrative, or myocardial storage diseases. STE helps in characterizing myocardial involvement patterns, acting as a sort of visual “fingerprint” and serving as an echo-based “tissue characterization tool” (Figure 3).^13^ There is a strong correlation between GLS values and late gadolinium enhancement (LGE) on cardiac magnetic resonance (CMR), pointing to myocardial fibrosis and higher risk of mortality and malignant VAs in patients with HCM.^14,15^ This parameter also has prognostic value in infiltrative diseases like CA.^16^

**Figure 3:** – Longitudinal strain patterns in left ventricular (LV) hypertrophic phenotype (bulls-eye parametric display). A) Hypertrophic cardiomyopathy (HCM) showing regional deformation alterations according to the distribution of hypertrophy (in this case, a septal asymmetric HCM), B) Cardiac amyloidosis, with a pattern of “apical sparing,” an echocardiographic red flag for its diagnosis, C) Fabry disease, with typical alteration of deformation in basal anterolateral wall, D) PRKAG2 cardiomyopathy, with massive biventricular hypertrophy in this case, with marked global alteration in GLS showing diffuse pattern, E) Friedreich’s ataxia, with concentric LV hypertrophy, showing alteration of deformation mainly in basal and medial segments, highlighting that “relative apical sparing” is not specific for the diagnosis of CA (this case: courtesy - Dr. Thiago Santos Rosa - Brazil).

Myocardial work has been developed as a new promising echocardiographic tool for the evaluation of myocardial mechanics, incorporating afterload (arterial blood pressure) as an estimate of LV pressure and using longitudinal strain (LS) for the construction of a “pressure x strain” loop, generated by a specific software.^17^ The additional value of this technique over conventional echocardiographic parameters for the evaluation of cardiomyopathies is still to be proved in larger studies. Still, some data are showing prognostic value for evaluation of HCM^18^ and CA^19^ for example.

While still not universally available, three-dimensional echocardiography (3DE) has become a valuable tool for assessing myocardial diseases, particularly for direct volumetric measurements of cardiac chambers. It provides accurate EF and LV mass values, correlating well with the gold standard CMR.^20^ 3DE also enables 3D myocardial strain measurement, is less affected by technical limitations like out-of-plane movement, and allows for the simultaneous evaluation of the entire LV, which is useful for synchronization analysis.

Contrast echo using ultrasound-enhancing agents is important for LV border delineation, especially in patients with suboptimal acoustic windows. It increases sensitivity in detecting conditions like apical HCM and apical aneurysms and differentiating intracavitary thrombus from other structures such as tendons or trabeculations.^21^

### Cardiovascular magnetic resonance

CMR has assumed an unquestionable role in the evaluation of LVH, mainly by assessing cardiac morphology, function, and tissue characterization.^8^ Although CMR may give essential clues for the final diagnosis in entities with extreme abnormalities in tissue characterization, such as CA or Fabry disease, the significant overlap of imaging findings in many entities makes necessary a comprehensive integration of imaging findings in the clinical context. No imaging finding should be interpreted in isolation, without integration of clinical history, electrocardiographic data and family history.^22^

Beyond the anatomical and functional evaluation of the LV, the main advantage of CMR compared to ECHO is the possibility of tissue characterization. Parametric mapping techniques that measure the T1 and T2 relaxation times have been increasingly incorporated in acquisition protocols, allowing for the quantitative evaluation of intracellular and extracellular components.^23^LGE imaging can identify replacement fibrosis and has well-established prognostic value, although it is less sensitive to detect diffuse interstitial collagen deposition than T1 mapping techniques.^24^

### Diagnosis of left ventricular hypertrophy

Despite the existence of established gender-specific normal reference values for LV mass in clinical guidelines,^25^ technical challenges can introduce variability and difficulties in the echocardiographic measurement of LV wall thickness. Suboptimal acoustic window, incorrect measurements (oblique or foreshortening, use of apical window – poor lateral resolution), and inclusion of confounding structures may affect accuracy. Some structures, such as a prominent RV moderator band, tricuspid valve apparatus, crista supraventricularis or LV fibromuscular false tendons inserted in the interventricular septum, may erroneously be interpreted as part of the septum, overestimating septal thickness. In pediatric patients, Z-scores, which represent the number of standard deviations from mean values, are used as reference standards. These scores adjust LV mass and wall thickness according to the child’s age and body size, providing a more tailored assessment in this population.^3^ Thresholds for carriers of genetic pathogenic variants may be lower^12^ and some presentations may cause confusion and misdiagnosis, such as late and already dilated phenotypes.^26^ To ensure an accurate diagnosis, it is essential to correlate these measurements with the clinical background, the presence of other associated structural cardiac diseases, GLS values, and diastolic function. In certain cases, CMR may be necessary to confirm the diagnosis.

### Hypertrophic cardiomyopathy

HCM is defined by an increased LV wall thickness of ≥ 15mm, or ≥ 13mm (in individuals with a positive genotype or relatives of HCM patients), in the absence of conditions that would justify secondary LVH such as severe AH, AS, or aortic coarctation, and excluding any infiltrative systemic diseases.^27^ It is crucial to recognize that HCM is not merely a myocardial disease; other features supporting the diagnosis include MV and subvalvular apparatus abnormalities like mitral leaflet elongation, papillary muscle hypertrophy, abnormal secondary MV chords, and muscle bundles (Figure 4).^8,28,29^

**Figure 4:** – Echocardiographic clues for the differential diagnosis of left ventricular hypertrophic phenotype. HCM: hypertrophic cardiomyopathy; SAM: systolic anterior motion of the mitral valve; MR: mitral regurgitation; RV: right ventricular.

Asymmetric septal LVH is the most classic pattern of HCM. Still, other phenotypic expressions like apical, concentric, lateral wall, midventricular, and less typical presentations involving any segment of the LV are common. The classical diagnostic criteria for diagnosing HCM, which are the same for both ECHO and CMR,^8^ have recently been brought into question (“one size fits all”) and in the future, probably gender, body surface and race will be considered for the definition of new thresholds. In particular, these criteria may not be reached in the apical variant, characterized by the loss or reversal of usual apical myocardial tapering. For instance, new cut-off values and diagnostic criteria were recently suggested for the detection of apical HCM, being the upper limit of normal apically 11 mm or 5.6 mm/m^2^. ^22,30^

RV hypertrophy is also frequent in HCM patients, found in 30-44% of cases, usually alongside LVH.^31,32^ RV dynamic obstruction may occur, either intraventricular or in the RV outflow tract.^33^ Conventional echocardiographic indices of RV systolic function, such as tricuspid annular plane systolic excursion (TAPSE), fractional area change (FAC), and tissue Doppler velocities, are typically normal. Still, subclinical systolic dysfunction may be identified through alterations in RV-LS.^34,35^

ECHO has also played an important role in the risk assessment and stratification of HCM patients,^36^ with studies showing higher mortality in HCM patients with LV septal thickness ≥ 30mm, apical aneurysm, or LV dysfunction (LVEF <50%).^3,37^ 2D LV GLS strongly correlates with fibrosis in HCM patients. Absolute GLS values and mechanical dispersion have a good correlation with the percentage of LGE and are independent predictors of VAs.^15^ In the study of Reant et al.,^14^ GLS absolute values < 15,4% were associated with heart failure, death and hospital admissions in a cohort of HCM patients. The parametric 2D LS bull’s eye plot derived from 2D STE may offer an intuitive visual overview of the global and regional LV myocardial deformation in HCM and is characterized by severely reduced segmental strain values in the most hypertrophied walls, usually more pronounced than other etiologies such as hypertensive or LVH secondary to AS.^38^

Hemodynamic evaluation to identify those with LVOT obstruction is crucial for treating HCM patients. Approximately one-third of HCM patients have rest LVOT obstruction (> 30 mmHg), with another third showing latent obstruction, revealed through bedside provocative maneuvers (Valsalva, standing, squatting-elevation, amyl nitrite inhalation) or exercise echocardiography.^39^Mitral regurgitation is a common finding in patients with HCM, especially in patients with systolic anterior mitral movement (SAM), and can be a major determinant of symptoms. It is very important to highlight that SAM is not a result solely of septal asymmetric hypertrophy (LVOT high velocities and Venturi effect). In fact, primary abnormalities of the MV apparatus, such as hypertrophy and anterior displacement of papillary muscles, leaflet elongation, and alteration in chordal insertion, may have a great role in LVOT obstruction (Figure 5).^28,40^

**Figure 5:** – Example of a patient with obstructive Hypertrophic Cardiomyopathy. A) septal thickness: 4,1cm, B) Longitudinal strain pattern (bulls-eye) showing regional alteration mainly in septal and segments, C) 3D transthoracic echo (TTE) acquisition, rendered 3D images longitudinal showing systolic anterior motion (SAM) of the mitral valve in systole (*), D) 3D TTE acquisition, rendered color 3D images (longitudinal view) showing mitral regurgitation secondary to SAM (*), E) Continuous Doppler showing late peak rest gradient in left ventricular outflow tract of 96mmHg, classic dagger shape pattern, F) 3D TTE acquisition, rendered 3D images (short axis view) showing papillary muscles anomaly with 4 heads (*) and anteriorly positioned, G) Cardiac MR showing massive septal hypertrophy, and in H- late gadolinium enhanced images showing septal fibrosis with midwall pattern.

HCM typically presents with normal or increased LVEF, the classic phenotype of HFpEF, associated with diastolic dysfunction in various degrees. Some patients may exhibit apical aneurysms and progressive LV dysfunction, which may lead to end-stage cardiac disease or the “burned-out” HCM variant, associated with a worse prognosis.^41,42^

CMR also allows for a detailed anatomical characterization regarding the pattern of LVH, variations in MV apparatus and their contributions to LVOT obstruction.^43^Considering this detailed evaluation, CMR plays an important role in planning septal reduction therapies.^44^

Regarding tissue characterization, native T1 and ECV correlate with diffuse fibrosis, elevated even in areas without LGE.^45,46^ Typical replacement fibrosis with a midwall pattern, more frequent in hypertrophic areas, is well depicted by LGE, with a well-recognized prognostic value.^47^Using recent advances, diffusion tensor acquisition allows the study of myocyte disarray, a premature marker of the disease,^48^ and stress perfusion CMR allows the study of microvascular dysfunction.^49,50^

### HCM Mimics

#### Cardiac amyloidosis

CA is an infiltrative CM caused by extracellular deposition of amyloid fibrils, with a classic phenotype of LVH (“pseudo-hypertrophy”) and HFpEF. ECHO, particularly in the early stages,^51^ lacks specificity to precisely distinguish amyloid from nonamyloid infiltrative or hypertrophic heart diseases, reinforcing the need to correlate with other clinical “red flags” and complement with other imaging modalities. Classical ECHO findings may be observed only in advanced stages of amyloid infiltration, with biatrial enlargement, valves and interatrial septal thickening, pleural and pericardial effusion, low myocardial velocities, and biventricular hypertrophy with a bright and sparkling appearance. Usually, these patients present with preserved LVEF and marked diastolic dysfunction with increased LV filling pressures (type II-III diastolic dysfunction), although reduced LVEF is a frequent finding in late-stage disease.^52,53^ It is important to notice that CA patients may present with reduced stroke volume even before a reduction in LVEF, caused by different factors, including alteration in myocardial deformation, impaired LV diastolic performance, atrial mechanic dysfunction and reduced LV volumes due to wall thickening.^54,55^

ECHO is a vital instrument for the early diagnosis of CA, particularly when encountering patients with the hypertrophic phenotype (defined as LV wall thickness ≥ 12mm) combined with other clinical or echocardiographic “red flags.” These findings (Figure 4) should prompt the clinician to direct these patients toward a specialized investigative pathway. This pathway typically includes CMR, scintigraphy with bone tracers, and the quantification of serum-free light chains, along with serum and urine immunofixation tests. These diagnostic modalities help in confirming the diagnosis of CA and differentiating monoclonal immunoglobulin light chain (AL) from transthyretin (ATTR) types, and also assess the staging of the disease and prognosis.^56^

GLS is notably impaired in patients with CA, demonstrating a strong correlation with the extent of amyloid burden, as shown in studies comparing GLS with LGE and extracellular volume (ECV) measured by CMR.^13,54^ A characteristic regional pattern of preserved longitudinal deformation in the apical segments, forming a basal-to-apical gradient or a relative apical sparing pattern (RASp), has been identified in CA (Figure 6). There are many ways to identify this pattern by STE, using different formulas and quantitative criteria or even considering a “cherry on top” visual appearance a qualitative visual sign derived from LS parametric (“bulls-eye”) analysis. RASp has shown good accuracy in distinguishing CA from other causes of LVH and myocardial diseases.^13,57,58^Although RASp is not specific for the diagnosis of CA,^59^ and may be found in other causes of LVH, it can be used as a valuable echocardiographic “red flag” to warrant further investigation in patients with a compatible clinical background,^60^ and also to determine prognosis in these patients.^61^ It is important to emphasize that RASp can be observed across different types of CA, including AL, hereditary ATTR, and wild-type ATTR amyloidosis, and is not helpful in distinguishing among them. This pattern may not be present in a significant proportion of patients because, in the initial stages of the disease, only mild grades of amyloid infiltration in basal segments may be present. On the other hand, a diffuse pattern of involvement of the myocardium may occur in the late-stage disease without a significant gradient between the apex and base of the heart.^62^ Considering the disproportionate and early drop in GLS and relatively preserved EF in patients with CA, the ratio of LVEF divided by GLS showed good accuracy in differentiating CA from HCM, with a cutoff of 4,1.^63^

**Figure 6:** – Example of a patient with Cardiac Amyloidosis. A) ECG with low voltage in frontal leads, first-degree atrioventricular block, and “pseudoinfarction” pattern in precordial leads, B) a clinical red flag of the ruptured biceps tendon, C) apical 4-chamber showing biatrial enlargement, thickening of interatrial septum and atrioventricular valves, concentric left ventricular (LV) hypertrophy, D) atrial strain with reduced reservoir component (+15%) usually caused by atrial myopathy and diastolic dysfunction, E) LV longitudinal strain (LS) bulls-eye (parametric display) showing altered regional strain in basal and medial segments, relatively preserved in apical segments (“apical sparing” or “cherry on top”). F) reduced right ventricular (RV) LS (free wall strain = -13%) showing infiltration of amyloid in RV wall, G) Cardiac MR showing biventricular wall thickening, interatrial septum, biatrial enlargement, H) Cardiac MR with late gadolinium enhancement showing subendocardial global pattern, with altered gadolinium kinetics.

RV myocardial deformation is typically impaired in patients with CA, which can be a helpful diagnostic feature in differentiating CA from other causes of hypertrophic phenotypes. Interestingly, a pattern of RV relative apical sparing, similar to that observed in the LV, has also been identified in these patients. The identification of this RV pattern, along with LV findings, enhances the diagnostic specificity for CA, as referenced in some publications.^64,65^

CMR is particularly useful for CA once the T1 values are notably extreme and LGE has typical kinetics^22^ (Figure 7). Amyloidotic myocardium has a singular avidity for the gadolinium leading to a myocardial “null point” earlier than the LV blood pool. As an extracellular contrast, gadolinium accumulates in the presence of extracellular space expansion secondary to amyloid deposition.^66^ LGE pattern is typically global and subendocardial, becoming transmural in advanced stages.^67^Due to the extracellular accumulation of amyloid fibrils, ECV is markedly increased, frequently higher than 40%.^68^

**Figure 7:** – Use of cardiac magnetic resonance (CMR) in patients with left ventricular (LV) hypertrophic phenotype, with EKG, cine CMR, late gadolinium enhancement (LGE) images and T1-sequence mapping. In A) a patient with asymmetric septal hypertrophic cardiomyopathy; in B) a patient with apical hypertrophic cardiomyopathy; C) a patient with ATTR cardiac amyloidosis (CA); D) a patient with Fabry’s disease. Arrows show areas of fibrosis, and a (*) points to a typical diffuse pattern in CA. Of note, T1 mapping shows an increased native T1 in a CA patient (C) and reduced T1 time in a Fabry patient (D).

When used alone, CMR does not allow an accurate distinction between AL and ATTR amyloidosis, although some features are more in line with each type: RV LGE is apparent in most patients with ATTR amyloidosis, but only in about 70% of patients with AL amyloidosis; LV mass and ECV are higher in ATTR while native T1 and T2 are higher in AL amyloidosis secondary to light chain toxicity in cardiomyocytes.^67,68^Furthermore, the calculation of ECV in the liver and spleen may identify systemic involvement in AL, which is very rare in ATTR.^69^

Combining multimodality imaging and observing their characteristics may provide important clues to identify potential differential diagnoses. Characteristic features of amyloidosis on CMR with grade 0 or 1 on bone scintigraphy point toward the diagnosis of AL amyloidosis, or rarely TTR variants, AApoAI and AApoAIV-amyloidosis.^70^ When combined with unremarkable monoclonal protein studies, the presence of characteristic features on CMR shows a high specificity for the diagnosis of ATTR CA.^71^

Fabry Disease

Fabry disease is a rare lysosomal storage disorder caused by a deficiency in α-GalA. LVH is the main cardiac manifestation of Fabry disease, and this entity accounts for 0.9% of the cases of HCM.^72^ In men, the chronic accumulation of globotriaosylceramide is responsible for 2% of the total cardiac hypertrophy, but its storage triggers sarcomeric protein expression via myocyte hypertrophy. On the other hand, in women, LVH consists of balanced sphingolipid and myocyte hypertrophy in proportion. It is often underdiagnosed and can lead to poor outcomes if left untreated. Cardiac involvement is the most crucial prognostic factor in Fabry disease and significantly impacts the quality of life.^6^ Cardiac alterations in Fabry disease may be subtle in young patients. Still, they typically develop HFpEF, arrhythmias, and LVH mimicking HCM later in life, generally after 30 years in men and 40 years in women. Early diagnosis is vital, especially because enzyme replacement therapy is available and can limit disease progression.^73^

The typical echocardiographic features of Fabry disease are concentric LVH with preserved EF and disproportionate hypertrophy of the papillary muscles (Figure 4). However, some patients may exhibit asymmetric LVH and even dynamic LVOT obstruction, leading to misdiagnosis as HCM. Dilatation of the aortic root and thickening of the mitral and aortic valves may occur, usually without significant dysfunction. RV hypertrophy with preserved systolic function is common in Fabry disease patients with LVH, and these patients usually exhibit better systolic function compared to CA patients with similar levels of RV wall thickening.^74^ GLS is significantly reduced in patients with overt Fabry disease, and regional strain alterations are often more pronounced in the basal inferolateral wall, correlating with LGE in this region on CMR. For carriers of pathogenic GLA gene variants, GLS facilitates early detection of cardiac involvement, independent of LVH.^75^

CMR shows native T1 characteristically low,^76^ reflecting the sphingolipid deposition. Once the storage is mainly an intracellular phenomenon, the extracellular space is spared by accumulation, resulting in a normal ECV measured by pre- and post-contrast T1.^76^ In fact, CMR may depict the three phases of the natural history of the disease. In the initial accumulation phase, a low native T1 is noted in the absence of LVH. The progression of the disease is documented by the appearance of hypertrophy, inflammation and LGE, mainly in the basal inferolateral wall. Finally, in the presence of extensive LGE there is a pseudonormalization of native T1.^76^

## Noonan Syndrome

Noonan syndrome is an autosomal dominant genetic disorder, part of a group known as RASopathies, which affects multiple body systems. It is characterized by a range of features, including congenital cardiac abnormalities, short stature, webbed neck, craniofacial dysmorphism, skeletal malformations, bleeding diathesis, hypertelorism, and mild intellectual disability. Mutations causing Noonan syndrome impact genes that encode proteins of the RAS-MAPK (mitogen-activated protein kinase) pathway. This leads to dysregulation of critical cellular processes, including proliferation, differentiation, survival, and metabolism, characteristic of RASopathies.

In over 80% of Noonan syndrome patients, cardiac abnormalities are observed, with pulmonary stenosis being prevalent in approximately 50% of cases and HCM occurring in 25%.^77^Additionally, Noonan syndrome is associated with a wide spectrum of other cardiac malformations.^78^LVH typically manifests early and is often diagnosed within the first six months of life.^79^LVH observed in Noonan syndrome can present as either concentric or asymmetric, sometimes accompanied by dynamic obstruction of the LVOT. MV anomalies and subvalvular complications, such as SAM, anomalous MV insertion leading to subaortic obstruction, and myxomatous degeneration resulting in valve prolapse, are commonly noted in Noonan syndrome patients with HCM.^80^ The presence of HCM in Noonan syndrome significantly influences patient outcomes, correlating with increased morbidity and mortality.^81^ The progression of LVH is variable; in some cases, it may emerge later in childhood and progress slowly, remain stable for several years, or rapidly evolve during infancy. In a subset of patients, representing 17% of a cohort of 46 subjects followed for seven years, regression and stabilization of LVH have been observed.^82^

## Pompe Disease

Pompe disease is classified as an autosomal recessive lysosomal storage disorder of rare incidence arising from mutations in the acid α-glucosidase gene. This genetic alteration results in an accumulation of lysosomal glycogen across various tissues, notably the myocardium, respiratory system, and skeletal muscles. The onset of Pompe disease varies, with a potential diagnosis occurring in infancy, childhood, or adulthood. The classic form of the disease, predominantly observed in infants, is characterized by rapid progression and typically presents with HCM, often prognosticating unfavorably. In such cases, untreated infants frequently succumb to cardiorespiratory failure within the first year of life. Phenotypically, Pompe disease is marked by LVH, predominantly with asymmetric septal thickening, although concentric hypertrophy involving both the septal and free walls of the LV and RV is also noted. In CMR, LGE is rare and can be seen in the subendocardium of the lateral and anterior walls. Severe septal hypertrophy often leads to SAM and LVOT obstruction, exacerbating clinical symptoms. These pathologies can progress to diastolic and systolic dysfunction, culminating in HF. Notably, enzyme replacement therapy has been observed to induce rapid regression of LVH and enhance systolic ventricular function, as assessed by myocardial deformation analysis.^83^

### PRKAG2 cardiomyopathy

PRKAG2 CM, an autosomal dominant glycogen storage disease that primarily affects the heart muscle and conduction system, presents with a unique clinical profile and prognosis. PRKAG2 CM is characterized by LVH, Wolff-Parkinson-White syndrome, and progressive conduction system disease.^84^HCM in patients with PRKAG2 gene mutations typically emerges in the teenage years or adulthood, with few cases reported in infancy. PRKAG2 CM is associated with worse outcomes compared to sarcomeric HCM, with patients potentially experiencing early cardiac failure and sudden death.^85^ The echocardiographic phenotype often shows concentric LVH, preserved LVEF, diastolic dysfunction, and, less commonly, RV hypertrophy^86,87^ (Figure 8). LVOT obstruction is rare in these patients, compared to patients with sarcomeric HCM. A study showed that patients with PRKAG2 CM may have more preserved GLS, associated with bradycardia, although having similar LV mass and thickening of ventricular walls.^88^

**Figure 8:** – Example of a young patient (25y old male) with PRKAG2 cardiomyopathy. In A) parasternal long axis view showing a remodeled left ventricle (LV) with hypertrophy. In B) EKG showing delta waves, short PR interval (Wolff-Parkinson-White syndrome) and LV hypertrophy pattern. In C) short axis view depicting concentric LV hypertrophy. In D) LV longitudinal strain (LS) bulls-eye (parametric display) showing diffuse alteration of myocardial deformation. E) biventricular concentric hypertrophy and an ICD inside right cavities. F) altered right ventricular longitudinal strain (medium free wall strain = -9,33%).

### Friedreich’s Ataxia

Friedreich’s ataxia is an autosomal recessive degenerative disease, affecting the frataxin gene, leading to mitochondrial iron storage and affecting glucose metabolism, the nervous system and the heart.^89^ Cardiac disease usually manifests as an HCM phenotype that may evolve into dilated cardiomyopathy, the most important cause of death.^90^Myocardial involvement may be detected subclinically before the development of LVH or LVEF reduction, either by STE^91^or CMR.^92^

Echocardiographic typical features are concentric LVH without LVOT or midventricular obstruction (although asymmetric hypertrophy may also occur), with LV dysfunction (reduced EF) and heart failure in advanced disease^93^with altered perfusion reserve.^8^ The morphological aspect of LVH in these patients may resemble CA, even with a sparkling granular texture of myocardium. Still, usually, there is no bi-atrial enlargement, pericardial effusion or severe diastolic dysfunction in patients with Friedreich’s ataxia.^94^

### Danon Disease

Danon disease is a rare X-linked dominant genetic disease that manifests with the clinical triad of cardiomyopathy, skeletal myopathy, and intellectual disability. It is caused by mutations in the lysosome-associated membrane 2 (LAMP2) gene. Danon cardiomyopathy is progressive and typically manifests as a hypertrophic phenotype, with alterations in radial, circumferential, and longitudinal strain in early stages, with preserved ejection fraction (HFpEF). With the progression of fibrosis, these patients may evolve with a decline in ejection fraction, worsening of symptoms and a dilated phenotype, particularly in male patients.^95^ The extent and severity of cardiomyopathy is the major prognostic factor. Most patients are asymptomatic during childhood, progressing to a symptomatic stage during adolescence and culminating in fulminant heart failure and sudden death in adulthood.^96^ CMR findings have a pivotal role in the diagnosis of this disease and typically include marked LVH, which may be concentric or asymmetric, and distinct patterns of LGE that differ from other forms of hypertrophic cardiomyopathy. LGE often spares the mid-septum and exhibits a base-to-apex gradient with the involvement of the apex.^97,98^ Furthermore, cardiac MRI can reveal elevated native T1 and ECV, suggesting myocardial fibrosis.^98,99^

## Conclusion

The association of the different pieces of the LVH puzzle (patient’s personal and family history, clinical presentation, physical examination findings, ECG features, and multimodality imaging data) can identify specific “red flags” that help the clinician differentiate between different causes of hypertrophic phenotypes.

This systematic approach allows for more accurate diagnoses and tailored management strategies. However, it is important to note that further diagnostic tests, such as genetic testing, cardiopulmonary exercise test and endomyocardial biopsy, may be necessary to confirm the underlying etiology. Clinical judgment and individualized patient assessment remain crucial in the diagnostic process.
